# Non-neoplastic diseases of the fallopian tube: MR imaging with emphasis on diffusion-weighted imaging

**DOI:** 10.1007/s13244-016-0484-7

**Published:** 2016-03-18

**Authors:** Pietro Valerio Foti, Noemi Ognibene, Saveria Spadola, Rosario Caltabiano, Renato Farina, Stefano Palmucci, Pietro Milone, Giovanni Carlo Ettorre

**Affiliations:** Radiodiagnostic and Radiotherapy Unit, University Hospital “Policlinico-Vittorio Emanuele”, Via Santa Sofia 78, 95123 Catania, Italy; Department G.F. Ingrassia – Institute of Pathology, University of Catania, Catania, Italy

**Keywords:** Fallopian tube, Magnetic resonance imaging, Pelvic inflammatory disease, Adnexal masses, Diffusion-weighted imaging

## Abstract

**Objective:**

We illustrate the magnetic resonance imaging (MRI) features of non-neoplastic tubaric conditions.

**Background:**

A variety of pathologic non-neoplastic conditions may affect the fallopian tubes. Knowledge of their imaging appearance is important for correct diagnosis. With recent advances in MRI, along with conventional MR sequences, diffusion-weighted imaging (DWI) sequences are available and may improve lesion characterization by discriminating the nature of the content of the dilated tube. Tubal fluid with low signal intensity on T1-weighted images, high signal intensity on T2-weighted images and no restricted diffusion on DWI is indicative of hydrosalpinx. Content with high signal intensity on T1-weighted images and restricted diffusion on DWI is suggestive of hematosalpinx associated with endometriosis or tubal pregnancy. A dilated tube with variable or heterogeneous signal intensity content on conventional MR sequences and restricted diffusion on DWI may suggest a pyosalpinx or tubo-ovarian abscess. We describe morphological characteristics, MR signal intensity features, enhancement behaviour and possible differential diagnosis of each lesion.

**Conclusion:**

MRI is the method of choice to study adnexal pelvic masses. Qualitative and quantitative functional imaging with DWI can be of help in characterization of tubaric diseases, provided that findings are interpreted in conjunction with those obtained with conventional MRI sequences.

***Teaching Points*:**

• *Nondilated fallopian tubes are not usually seen on MR images.*

• *MRI is the method of choice to characterize and localize utero-adnexal masses.*

• *MRI allows characterization of lesions through evaluation of the fluid content’s signal intensity.*

• *DWI in conjunction with conventional MRI sequences may improve tissue characterization.*

• *Pelvic inflammatory disease is the most common tubal pathology.*

## Introduction

A wide variety of pathologic non-neoplastic conditions may affect the fallopian tubes, including common conditions such as pelvic inflammatory disease (PID), or uncommon ones such as isolated tubal torsion. Knowledge of the embryologic development and normal anatomy of the fallopian tubes and of the imaging appearances of these entities is important for correct diagnosis and differentiation from other pathological entities. Multiplanar magnetic resonance imaging (MRI) can be particularly useful in characterizing female pelvic disease, because it is capable of identifying normal ovaries, demonstrating the tubular C- or S-shaped cystic nature of tubaric lesions, and differentiating pyosalpinx from hematosalpinx by characterizing the signal intensity of the tubal fluid [1, 2]. With recent advances in ultrafast MR imaging techniques, perfusion-weighted (PW) and diffusion-weighted imaging (DWI) are available to assess microvascular and cellular characteristics in abdominal and pelvic organs [3, 4]. Diffusion-weighted imaging is an evolving technology with the potential to improve tissue characterization when findings are interpreted in conjunction with those obtained with other conventional MR imaging sequences [5]. Diffusion-weighted imaging performed with parallel imaging technique has indeed demonstrated potential for differentiating benign from malignant gynaecologic lesions. Apparent diffusion coefficient (ADC) measurements can help differentiate between normal and cancerous tissues; however, this usefulness may be limited by significant overlap between ADC values in normal and abnormal tissue [6–11].

In this article we review embryologic, anatomical, and histological features of fallopian tubes. We try to familiarize the radiologist with the variable MR imaging appearance of different non-neoplastic tubaric conditions by describing morphological characteristics, MR signal intensity features (e.g. fluid, hemorrhagic, purulent content), and enhancement behaviour of each lesion using examples pathologically proven from our institution; possible differential diagnosis are proposed from time to time. Our MR protocol is also included, with emphasis on diffusion-weighted imaging.

### Embryologic features

**Fig. 1:**
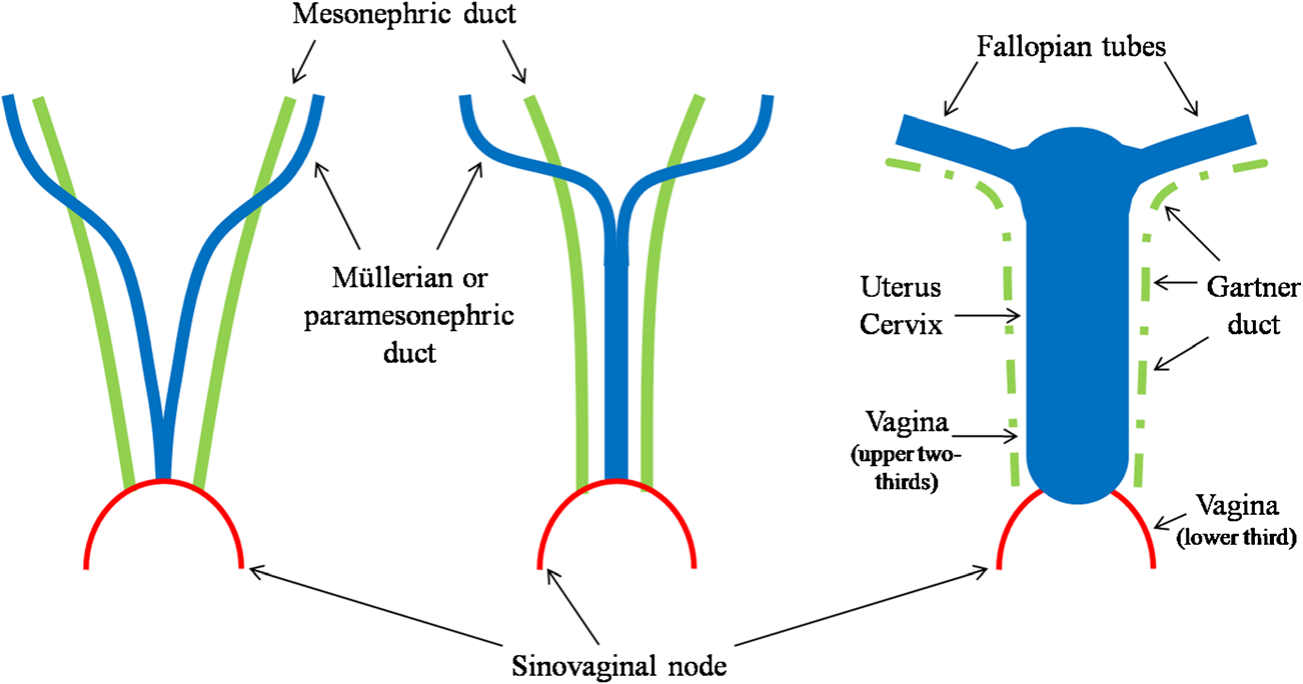
Embryologic development of the gynaecological tract. Diagram illustrates the embryologic development of the gynaecological tract. Between the fifth and sixth week after oocyte fertilization, a longitudinal groove called Müller’s groove arises from the coelomic epithelium on each lateral side to the mesonephric duct; the edges of this groove fuse to form a canal called the Müllerian or paramesonephric duct. Three parts of the paramesonephric ducts are recognizable: a cranial vertical portion, which opens directly into the coelomic cavity; a middle horizontal part, which crosses the mesonephric duct; a caudal vertical part, which fuses with its partner from the opposite side. In the eighth week the paired paramesonephric ducts fuse to form the upper two-thirds of vagina, the cervix, uterus, and both fallopian tubes. The lower third of the vagina is formed as the sinovaginal node canalizes. The mesonephric ducts regress as Gartner’s duct

Knowledge of the normal development and anatomy of the gynaecological tract is critical to understand the nature of a variety of gynaecological diseases. Between the fifth and sixth week after oocyte fertilization, a longitudinal groove called Müller’s groove arises from the coelomic epithelium on each lateral side to the mesonephric duct. The edges of this groove fuse to form a canal called the Müllerian or paramesonephric duct (Fig. 1) [12]. The paramesonephric ducts are the precursors of the uterus, fallopian tubes, cervix and upper vagina [13]. Three parts of the paramesonephric ducts are recognizable during this process: a proximal (cranial) vertical portion, which opens directly into the coelomic cavity; a middle (horizontal) part, which crosses the mesonephric duct; and a distal (caudal) vertical part, which fuses with its partner from the opposite side [14]. In the eighth week, the paired paramesonephric ducts fuse to form the upper two-thirds of vagina, the cervix, uterus, and both fallopian tubes. The cranial end of the fused ducts yields the future uterus, which contains mesoderm that will form the uterine endometrium and myometrium. The unfused cranial ends of the paramesonephric ducts assume a funnel-shaped configuration and remain open to the future peritoneal cavity as the fimbrial portions of the fallopian tubes. The caudal end of the fused ducts will form the upper two-thirds of the vagina. The lower third of the vagina is formed as the sinovaginal node (bulb) canalizes [13].

### Anatomic features

The eponymous name—Fallopian tube—is named after Gabriel Fallopius: Italian anatomist (1523–62), the same anatomist who gave his name to the Fallopian ligament and the Fallopian canal [15]. The uterine tubes are paired, tubular uterine appendages located bilaterally at the superior portion of the uterine cavity. These tubes exit the uterus through an area referred to as the cornua, forming a connection between the endometrial and peritoneal cavities. The Fallopian tubes situated in the upper margins of the broad ligaments between the round and utero ovarian ligaments. The primary function of the uterine tubes is to transport sperm toward the egg, which is released by the ovary, and to then allow passage of the fertilized egg back to the uterus for implantation [16].

**Fig. 2:**
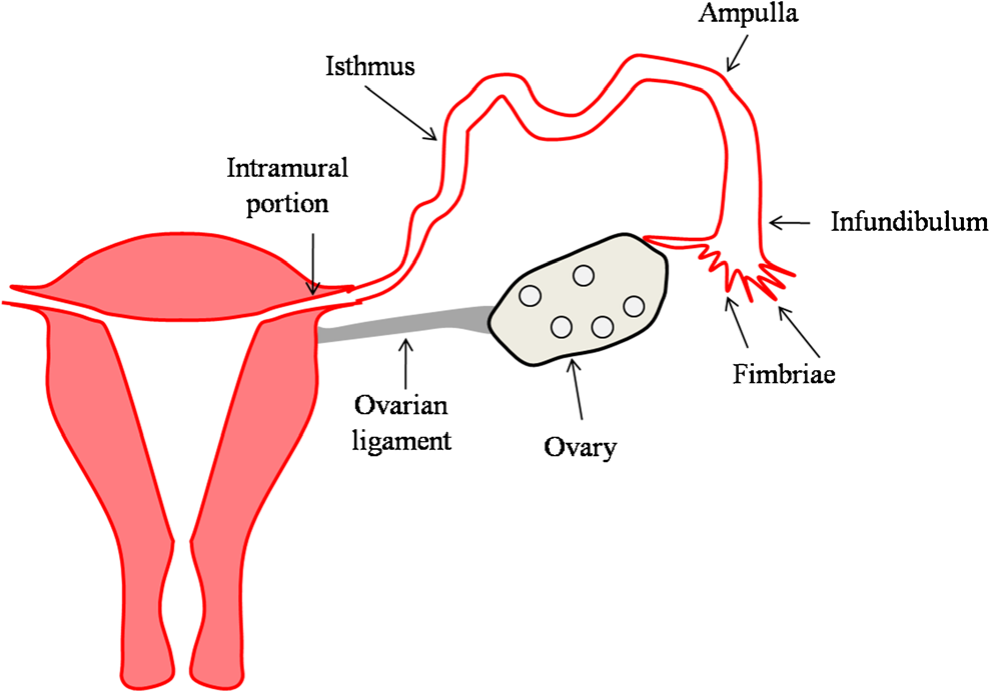
Anatomy of Fallopian tube. Diagram illustrates the normal anatomy of Fallopian tube. The uterine tube has been distinguished in four anatomical regions: infundibulum with fimbriae, which are closely associated with the ovary, that opens into the peritoneal cavity; ampulla with highly folded structure with plicae and secondary folds dividing the lumen; isthmus with fewer mucosal folds and a thick muscularis layer; intramural portion that passes through the muscular wall of the uterus

Each tube is about 10 cm long [12]. In the adult, the uterine tube has been distinguished in four anatomical regions (Fig. 2):

- Infundibulum—the funnel-shaped open end of the uterine tube with fimbriae, which are closely associated with the ovary, that opens into the peritoneal cavity by its abdominal ostium;
- Ampulla—uterine tube with highly folded structure with plicae (mucosal folds) and secondary folds dividing the lumen; usual site for fertilization;
- Isthmus—narrow portion of the uterine tube with fewer mucosal folds and a thick muscularis layer;
- Intramural—uterine tube that passes through the muscular wall of the uterus [17].

The tubal lumen varies in width along its length, measuring approximately 1 mm in the pars interstitialis to 4 mm in the ampulla/infundibulum [14].

### Histological features

The tubal wall consists of three layers: the internal mucosa (endosalpinx), the intermediate muscular layer (myosalpinx), and the outer serosa, which is continuous with the peritoneum of the broad ligament and uterus, the upper margin of which is the mesosalpinx. The endosalpinx is thrown into longitudinal folds, called primary folds, increasing in number toward the fimbria and lined by columnar epithelium of four cellular types [17]. The ciliated cells have fine granular cytoplasm and are relatively square with large round nuclei. Secretory cells or nonciliated cells have a heavily granular cytoplasm and an oval nucleus. The intercalary or “placed-between” cells are long narrow cells with dark nuclei causing them to be called “peg cells.” The fourth types of cells are the small “indifferent” cells with large dark nuclei [18]. In the ampullary and infundibular sections, secondary folds of the tubal mucosa also exist, markedly increasing the surface areas of these segments of the tube [17]. Secretory activity varies during the menstrual cycle, and resting secretory cells are also referred to as peg cells. Some of the secreted substances are thought to nourish the oocyte and the very early embryo [19]. The myosalpinx consists of an inner circular and an outer longitudinal layer to which a third inner longitudinal layer is added in the isthmus and the intramural portion of the tube [17]. Peristaltic muscle action seems to be more important for the transport of sperm and oocyte than the action of the cilia [19]. Muscle is sensitive to sex steroids, and thus peristalsis is greatest when oestrogen levels are high [20]. The serosa of the tube is composed of an epithelial layer that is histologically indistinguishable from peritoneum elsewhere in the abdominal cavity [17].

## Our MR protocol

The MR imaging protocol we use in our institution to study patients with adnexal masses or suspected fallopian tube disease is as follows.

MR imaging is performed with a closed-configuration superconducting 1.5-T system (Signa HDxT; GE Healthcare, Milwaukee, Wis) with 57.2 mT/m gradient strength and 120 T/m/s slew rate, by using an eight-channel high-resolution torso coil with array spatial sensitivity technique (ASSET) parallel acquisition.

### MR sequences

- Localiser sequence in the three spatial planes;
- Axial T2-weighted single-shot fast spin-echo (SSFSE) sequence (time to repetition (TR)/time to echo (TE) range 765/59; flip angle 90°; section thickness 6 mm; interslice gap 0.6 mm; bandwidth 31.25 kHz; field of view (FOV) 38 cm; matrix 320 × 288; number of averages 0.54; number of images 30; acquisition time 24 s) used as second localiser to identify the longitudinal axis of the uterus in the case of laterally deviated uterus;
- Sagittal T2-weighted fast spin-echo (FSE) sequence parallel to the longitudinal axis of the uterus (identified on the previous SSFSE sequence) (TR/TE range 4,675/100; flip angle 90°; section thickness 4 mm; interslice gap 1 mm; bandwidth 41.67 kHz; FOV 32 cm; matrix 320 × 224; number of averages 4; number of images 26; acquisition time 3 min 49 s);
- Oblique coronal T2-weighted FSE sequence parallel to the longitudinal axis of the uterus (TR/TE range 4,675/100; flip angle 90°; section thickness 4 mm; interslice gap 1 mm; bandwidth 41.67 kHz; FOV 32 cm; matrix 320 × 224; number of averages 4; number of images 26; acquisition time 3 min 49 s);
- Oblique axial T2-weighted FSE sequence perpendicular to the longitudinal axis of the uterus (TR/TE range 4,675/100; flip angle 90°; section thickness 4 mm; interslice gap 1 mm; bandwidth 41.67 kHz; FOV 32 cm; matrix 320 × 224; number of averages 4; number of images 26; acquisition time 3 min 49 s);
- Sagittal or oblique axial or oblique coronal fat suppressed T2-weighted FSE sequence (TR/TE range 4,675/100; flip angle 90°; section thickness 4 mm; interslice gap 1 mm; bandwidth 41.67 kHz; FOV 32 cm; matrix 320 × 224; number of averages 4; number of images 24; acquisition time 3 min 49 s);
- Axial T1-weighted gradient-echo (GRE) sequence in-out phase (chemical-shift imaging) (TR/TE 180/2,1; flip angle 80°; section thickness 6 mm; interslice gap 0,6 mm; bandwidth 62,5 kHz; field of view 38 cm; matrix 256 × 224 ; number of averages 1; number of images 20; acquisition time 22 s);
- Axial DWI SE EPI (TR/TE 3000/74,1; flip angle 90°; section thickness 5 mm; interslice gap 1 mm; bandwidth 250 kHz; field of view 45 cm; matrix 160 × 160; number of averages 16; number of images 14; b-value 0 e 800 s/mm^2^; acquisition time 1 min e 40 s);
- Sagittal, oblique coronal, oblique axial T1-weighted 3D gradient-echo liver acquisition with volume acquisition (LAVA) sequence with fat suppression (TRe/TE range 4.4/2.1; flip angle 12°; section thickness 3.4 mm; overlap locations −1.7 mm; bandwidth 62.5 kHz; FOV 40 cm; matrix 320 × 192; number of averages 0.75; number of images 104; acquisition time 22 s).

After intravenous (i.v.) administration of 0.1 mmol/kg paramagnetic contrast agent (Dotarem, Guerbet, Roissy, France) at a flow rate of 2 ml/s, followed by 20 ml of saline solution at the same flow rate, the following sequences are acquired:

- Dynamic sagittal T1-weighted 3D gradient-echo LAVA with fat suppression (TR/TE range 4.4/2.1; flip angle 12°; section thickness 3.4 mm; overlap locations −1.7 mm; bandwidth 62.5 kHz; FOV 40 cm; matrix 320 × 192; number of averages 0.75; number of images 104; acquisition time 22 s) acquired at 60 and 120 s after contrast administration;
- T1-weighted 3D gradient echo LAVA fat-suppressed sequence, in the oblique coronal (parallel to the longitudinal axis of the uterus), oblique axial (perpendicular to the longitudinal axis of the uterus) and axial planes (TR/TE range 4.4/2.1; flip angle 12°; section thickness 3.4 mm; overlap locations −1.7 mm; bandwidth 62.5 kHz; FOV 40 cm; matrix 320 × 192; number of averages 0.75; number of images 104; acquisition time 22 s).

When images suggest a dilatation of the urinary excretory system, the following sequences are acquired about 10 min after contrast administration to obtain an urographic phase:

- T1-weighted 3D gradient echo LAVA fat-suppressed sequence, in the sagittal, coronal and axial planes (TR/TE range 4.4/2.1; flip angle 12°; section thickness 3.4 mm; overlap locations −1.7 mm; bandwidth 62.5 kHz; FOV 40 cm; matrix 320 × 192; number of averages 0.75; number of images 104; acquisition time 22 s).

MR imaging is performed with the patient lying in the supine position (feet first), except for the urographic phase acquired in the prone position. T2-weighted FSE sequences and DW sequences are acquired with patient breathing freely, T1-weighted 3D gradient echo LAVA fat-suppressed sequence are acquired in breath hold.

T2-weighted sequences are useful to study the zonal anatomy of the uterus and its appendages, to identify and characterize most of the pathological processes and to visualize effusion in the pelvic cavity.

In-phase and out-of-phase T1-weighted sequences are useful to recognize a lesion within the presence of iron deposition or fat and to differentiate lesions with intracellular fat content (hypointense) from those with hemorrhagic content (hyperintense) such as endometriotic implants.

T1-weighted 3D gradient-echo LAVA sequences with fat suppression allow increases in spatial resolution without a significant time penalty; those acquired with breath holding after injection of the gadolinium chelate allow assessment of the vascularization of pelvic lesions.

DWI allows identification of neoplastic and inflammatory lesions as areas of low diffusivity compared to healthy tissue because of their high cellularity. The lesions showing restriction of diffusivity are hyperintense on DWI sequences and hypointense on ADC maps. DWI sequences are also helpful when impaired renal function contraindicates the use of imaging contrast agent.

## Technical considerations on diffusion-weighted imaging

### Principles of diffusion-weighted MR imaging

Diffusion-weighted imaging is an emerging noninvasive technique in body imaging that provides indirect information about the microenvironment of tissues and lesions. It can help to detect, characterize, and perform follow-up of tumours, and allows detection and grading of liver fibrosis and cirrhosis, detection of abscesses, and evaluation of inflammatory bowel disease.

Diffusion-weighted imaging in the abdomen and pelvis has been increasingly used since the 1990s with the development of stronger diffusion gradients, faster imaging sequences, and improvements in technology and MRI instrumentation [5]. Diffusion-weighted imaging derives its image contrast from differences in the motion of water molecules in various tissues. The degree of restriction to water diffusion in biologic tissue is inversely correlated with cellular density and the integrity of cell membranes. The motion of water molecules is more restricted in tissues with high cellular density and intact cell membranes (inflammatory foci, tumour tissue); in areas of low cellular density, or where the cell membrane has been breached, the motion of water molecules is less restricted [21]. Tissue types that have been reported to be associated with impeded diffusion include tumour, cytotoxic oedema, abscess, and fibrosis. Tissues with low cellularity or that consist of cells with disrupted membranes permit greater movement of water molecules [5]. DWI also has the capability to detect inflammatory foci [22, 23].

The normal ovaries and testes show restricted diffusion. This property can be used to identify the ovaries in girls with pelvic masses [24]. DWI can be used to assess pelvic inflammatory disease. Abscesses, including tubo-ovarian abscess, show restricted diffusion [25].

### Measurement of diffusion

A diffusion-weighted sequence was initially described by Stejskal and Tanner in 1965 [26] as an adaptation of a T2-weighted sequence. The clinical sequence commonly used is an ultrafast spin-echo-planar T2-weighted sequence with parallel imaging. For over 20 years, this technique has been used for brain imaging. Until the past decade, however, various applications in the study of the abdomen have been successfully developed, with the advancement of phased-array surface coils, high gradient amplitudes, and rapid imaging techniques (echoplanar imaging and parallel imaging) [21, 27, 28].

The diffusion sensitivity is easily varied by changing the parameter known as the *b* value. The term *b* value refers to the strength of the diffusion sensitizing gradient. The *b* value is proportional to the gradient amplitude, the duration of the applied gradient and the time interval between paired gradients, and is measured in seconds per square millimeter [29]. The sensitivity of the diffusion sequence is adjusted by varying the *b* value, which is most readily achieved by altering the gradient amplitude [21]. Water molecules with a high degree of motion or a great diffusion distance (within the intravascular space) will have decreased signal at low *b* values (b = 50–100 sec/mm^2^); thus, diffusion-weighted data acquired over a range of low *b* values have decreased signal due to perfusion. Conversely, high *b* values (*b* = 800 sec/mm2) are usually required to perceive slow-moving water molecules or small diffusion distances, both of which show more gradual decreases in signal with increasing *b* values.

### Quantitative analysis of diffusion-weighted imaging

The apparent diffusion coefficient (ADC), the quantitative parameter of DWI, represents the slope (gradient) of a line that is produced when the logarithm of relative signal intensity of tissue is plotted along the y-axis versus *b* values along the x-axis, thereby linearizing the exponential decay function. The analysis of ADC is an automated process that is available as an application on most scanners or on a workstation. Calculation of ADC is independent of magnetic field strength and is made for each pixel of an image. The ADC can be displayed as a parametric map and essentially reflects differences in tissue diffusivity at different b values. For ADC measurement regions of interest (ROIs) are drawn manually on the ADC map based on the corresponding axial T2-weighted images, and the oblique high-resolution T2-weighted images are used to identify the lesion. To avoid pitfalls in image interpretation, DWI and the derived ADC maps must be evaluated in conjunction with morphologic images. Since DW images are derived from T2-weighted images, tissues with a long relaxation time, such as simple cysts, can have high signal intensity on DW images, the so-called T2 shine-through effect [21].

### MR imaging at 3.0 Tesla

The role of 1.5 T MRI in the assessment of adnexal pathologic conditions has been widely established, but only in recent years 3.0 T MR systems have been applied in the study of gynaecologic diseases.

As for the magnetic field strength, the main advantage of 3.0 T MR systems is the expected increase in MR signal-to-noise ratio (SNR) that scales linearly with the field-strength (*B*_*0*_); this gain in SNR can be used to reduce the voxel size for high-resolution imaging, to reduce the acquisition time or a combination of both [30]. Nevertheless, pulse sequence parameters at 3.0 T need to be re-optimized from 1.5 T values in order to maintain desired image contrast. Image artefacts due to changes in tissue susceptibility, chemical shift, radiofrequency effects, and/or pulse sequence physics may be more noticeable and harder to suppress at 3.0 T [31].

In MRI of the female pelvis, increased spatial resolution feasible at 3.0 T provides clinically relevant information and allows the clinician to visualize and to classify pathology (e.g. identification of very small structures such as septa and nodules in the differential diagnosis and correct classification of cystic ovarian tumours) [32–34]. Short acquisition time constitutes a substantial clinical advantage of 3.0 T pelvic MRI since it allows the reduction of bowel motion-related artefacts and achievement of multiple anatomical views while maintaining or still decreasing overall acquisition time [32–34].

Compared with 1.5 T, the increased SNR and improved background suppression at 3.0 T may also allow better categorization of variable components (fluid, blood, etc.) [35].

3.0 T MR systems are theoretically ideal for DWI because they can provide twice the intrinsic SNR than those at 1.5 T [36]. Previous studies [37] demonstrated that protocol adjustment at 3.0 T is mandatory because when body DW-MRI protocols are transferred from 1.5 T to 3.0 T without further modifications, image quality may be seriously degraded and images can suffer from severe distortions and signal losses, thus making qualitative assessment and quantitative analysis difficult. In particular, DWI sequences can suffer from increased warping and susceptibility artefacts at 3.0 T [31]. In abdominal and pelvic DWI the images can be greatly distorted, especially at the body surface and at soft tissue interfaces with the air in the gastrointestinal tract [36]. Saremi et al. [36] demonstrated a considerable reduction in susceptibility and ghost artefacts after application of parallel imaging and breath hold techniques to a single-shot spin-EPI DWI sequence commonly used in practice. As for quantitative analysis, the Authors found no significant difference in the ADC values of the normal solid organs between 3.0 T and 1.5 T magnetic field strengths [36].

## Imaging findings

Nondilated fallopian tubes are not usually seen on MR images unless they are outlined by pelvic fluid [38]. In the presence of peritoneal fluid or contrast material, the fallopian tubes appear as paired, thin, serpentine juxtauterine structures extending either anteriorly or posteriorly into the cul-de-sac [39]. Serous fluid, haemorrhage, or pus may accumulate within the tube depending on the cause of the tubal obstruction and dilatation [40].

Pelvic inflammatory disease (PID) is inflammation of the upper genital tract and is the most common tubal pathology. PID is described as a spread of inflammation from the endometrial cavity and fallopian tubes into the pelvis [41]. The disorder may coexist with endometritis or oophoritis, may spread as peritonitis, and may extend along the paracolic gutters to the liver to cause the Fitz-Hugh-Curtis syndrome. It usually affects women in the reproductive age group and accounts for 25 % of visits to the emergency departments with gynaecological pain [41]. Consequences of PID include blockage of the tube usually either proximately, at the site of insertion into the uterus, or distally, causing a hydrosalpinx with partial or complete distal obstruction. Other sequelae may include pyosalpinx, tubaric or tuboovarian abscess, and peritubal adhesions. The long-term consequences of PID include recurrent PID in almost 25 % of cases after one episode of salpingitis, chronic pelvic or abdominal pain in one of every five affected patients, tubo-ovarian abscess in about 34 % of hospitalized patients, Fits-Hugh-Curtis syndrome and deep dyspareunia in two of every five patients, and menstrual disturbances in four of every five patients [12].

Table 1 summarizes MR imaging features of fallopian tube diseases along with the main differential diagnosis.

**Table 1:**
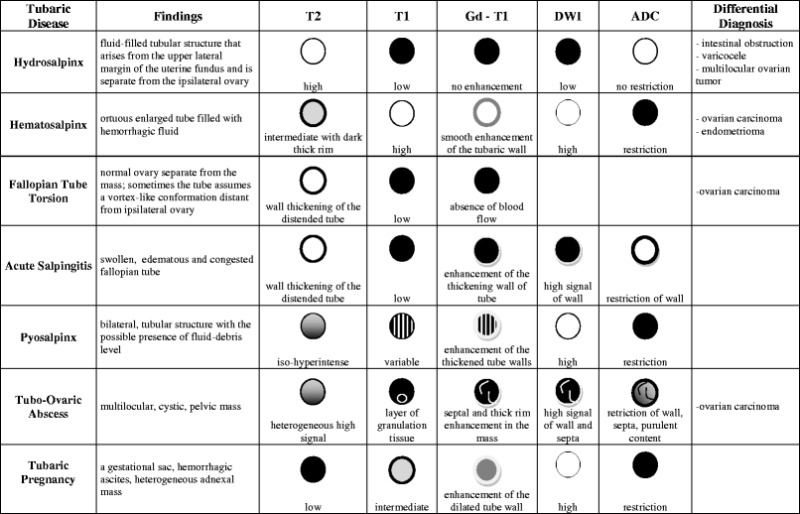
Chart summarizing MR imaging features of tubaric disease

*DWI* = diffusion-weighted MRI; *ADC* = apparent diffusion coefficient

The *hydrosalpinx*, common adnexal lesion found on imaging, is due to an expansion of the fallopian tubes due to obstruction of the ampullary segment that may occur either in isolation or as a component of a complex pathological process (PID, tubal ligation, hysterectomy, endometriosis, fallopian tube tumour, or tubal pregnancy). Repeated episodes of PID and subsequent formation of adhesions are the most common cause of tubal occlusion and hydrosalpinx.

**Fig. 3:**
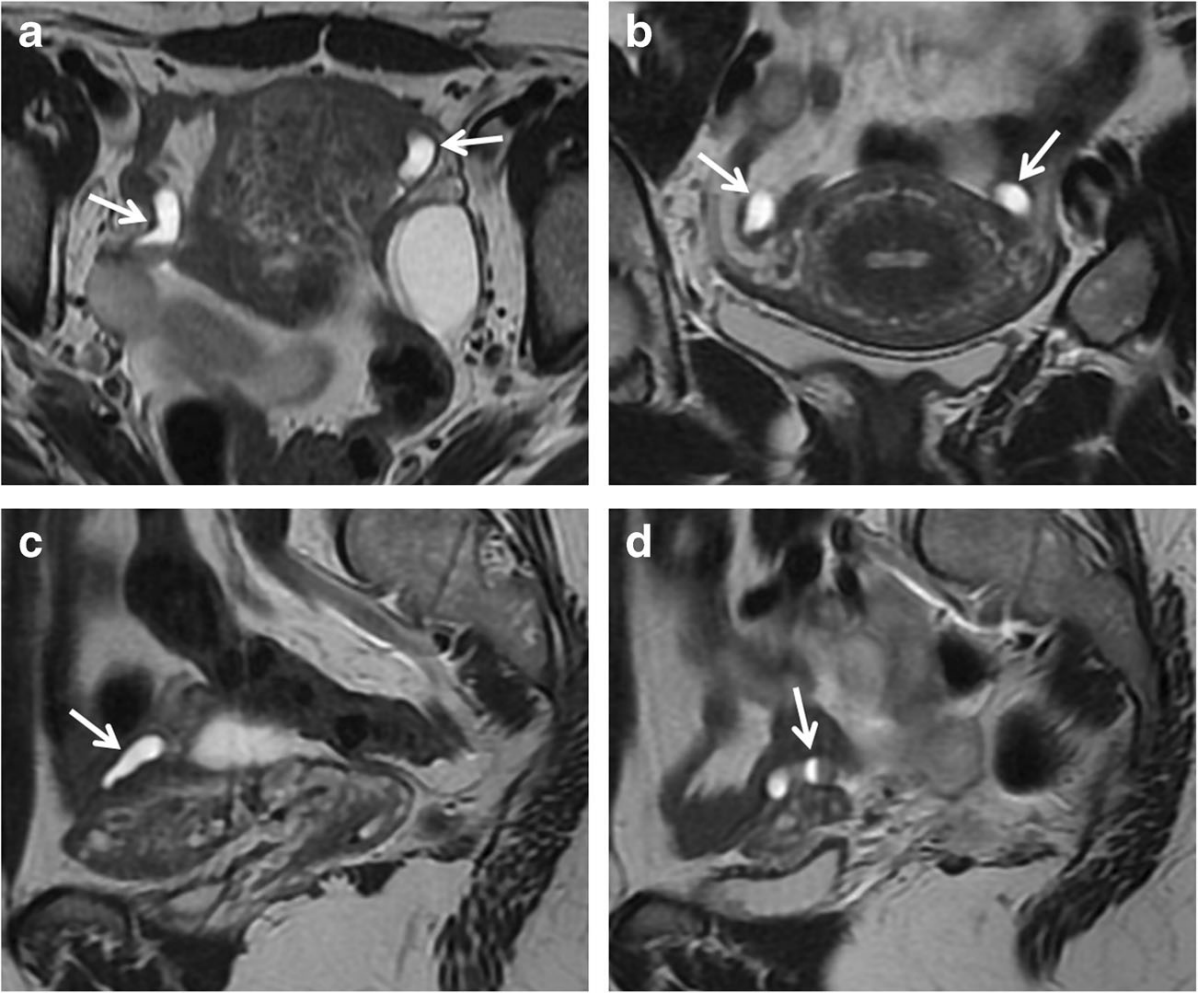
Hydrosalpinx in a 47-year-old woman who underwent tubal ligation. (**a**) Axial, (**b**) coronal, and (**c**, **d**) sagittal T2-weighted MR images show a paired, serpentine juxtauterine structure located bilaterally at the superior portion of the uterus with hyperintense signal and a fluid-fluid level content (*white arrows*)

Tubal sterilization does appear to be a risk factor for subsequent hydrosalpinx formation, whether or not the patient undergoes subsequent hysterectomy [42]. Women who have had PID or who have used intrauterine devices (IUDs) might be at risk of developing this condition because they may already have occluded tubes from prior salpingitis. If a previously occluded tube is ligated or cauterized so that a second occlusion is created, hydrosalpinx may be anticipated [43] (Fig. 3).

Even though classic teaching has stated that women who have undergone bilateral tubal ligation (BTL) are not susceptible to PID, its presentation in the setting of a prior BTL occurs with surprising frequency and deserves further study [44]. The majority of cases of salpingitis after previous tubal occlusion develop more than a year after either laparoscopic or laparotomy sterilization procedures. For tubo-ovarian abscess after previous tubal occlusion, this time interval ranges from several weeks to almost two decades. Most cases of salpingitis show inflammation of each tubal segments; when only one segment is involved, it is generally the proximal segment [45].

MRI is the method of choice for characterization and localization of utero-adnexal pelvic masses of uncertain nature. On MR images, the hydrosalpinx appears as a fluid-filled tubular structure that arises from the upper lateral margin of the uterine fundus and is separate from the ipsilateral ovary. A dilated fallopian tube folds upon itself to form a sausage-like C- or S-shaped cystic mass [1, 2]. The most helpful and specific findings of hydrosalpinx are a tubular shape with small round projections and the waist sign [46]. The dilated tubes contain incomplete plicae or folds, producing its convoluted appearance, either seen as the waist sign causing focal constriction of the tubular structure [47, 48].

**Fig. 4:**
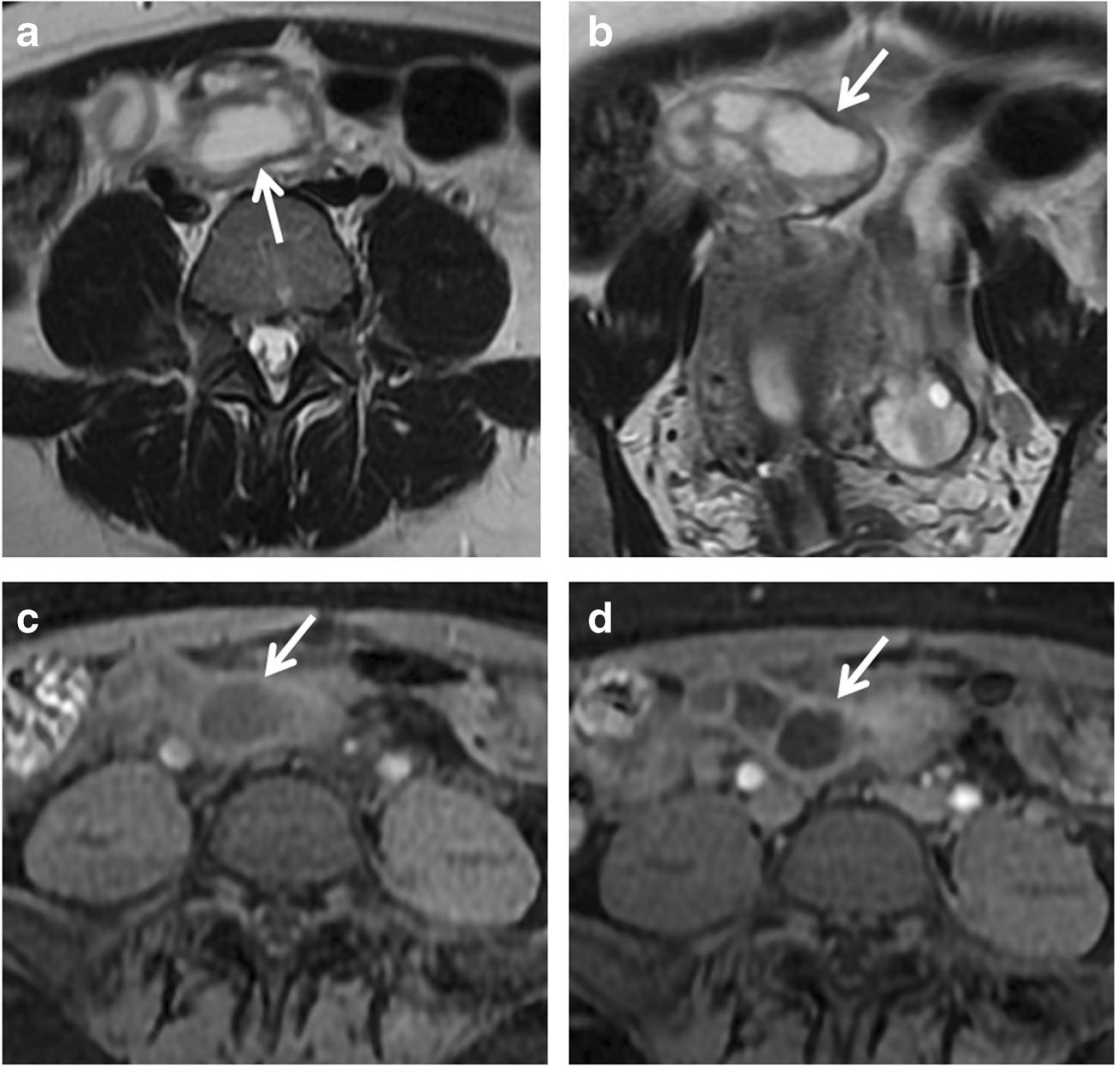
Hydrosalpinx in a 27-year-old puerperal woman. (**a**) Axial and (**b**) coronal T2-weighted MR images show a tortuous, tubular right adnexal structure with hyperintense content (*white arrows*). (**c**) Axial unenhanced and (**d**) contrast-enhanced fat-suppressed T1-weighted images show dilated tube with hypointense content and minimal parietal enhancement after contrast administration (*white arrows*)

**Fig. 5:**
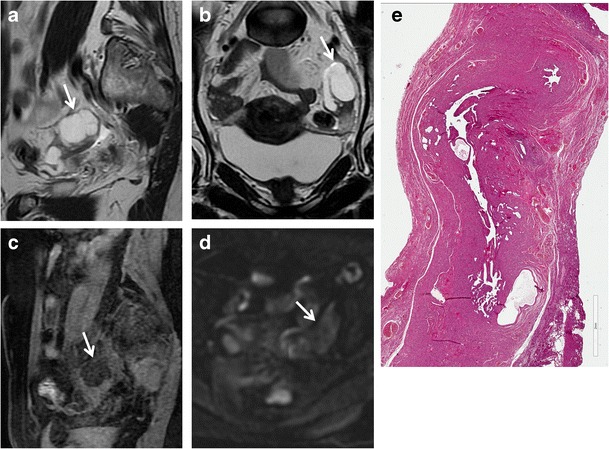
Hydrosalpinx in a 57-year-old woman who has been using intrauterine devices (IUDs) for about 20 years. (**a**) Sagittal and (**b**) coronal T2-weighted MR images show a distended left fallopian tube with high signal intensity content (*white arrows*). (**c**) Sagittal fat-suppressed T1-weighted image shows a distended fallopian tube with low signal intensity content (*white arrow*), a finding consistent with hydrosalpinx (**d**) Axial DW image (b = 800 s/mm^2^) shows that the lesion’s content does not demonstrate restricted diffusion with low signal intensity (*white arrow*). (**e**) Haematoxylin and eosin stained sample showed flattened tubal epithelium and fibrosis of the wall (H&E 100X)

MR imaging also allows the characterization of the lesion through the evaluation of the signal intensity of the fluid content. The hydrosalpinx appears hypointense on T1-weighted images and hyperintense on T2-weighted images (Fig. 4). The content has no restriction of diffusion on DWI sequence (Fig. 5). The hydrosalpinx enter into the differential diagnosis with intestinal obstruction and varicocele. If it reaches a diameter of 10–12 cm, hydrosalpinx may also simulate a multilocular ovarian tumour [49]. However, malignant tumours are generally depicted as foci of increased intensity on DW images because water diffusion is restricted in highly cellular tissues as malignant tumours [50, 51]. Anyway, some well-differentiated tumours may exhibit little restriction of diffusion due to their low cellularity. In contrast, blood, fat, abscesses, lymph nodes, and the melanin may show restricted diffusion. In these cases, reference to standard T1- and T2-weighted images can lead to the correct diagnosis [52].

**Fig. 6:**
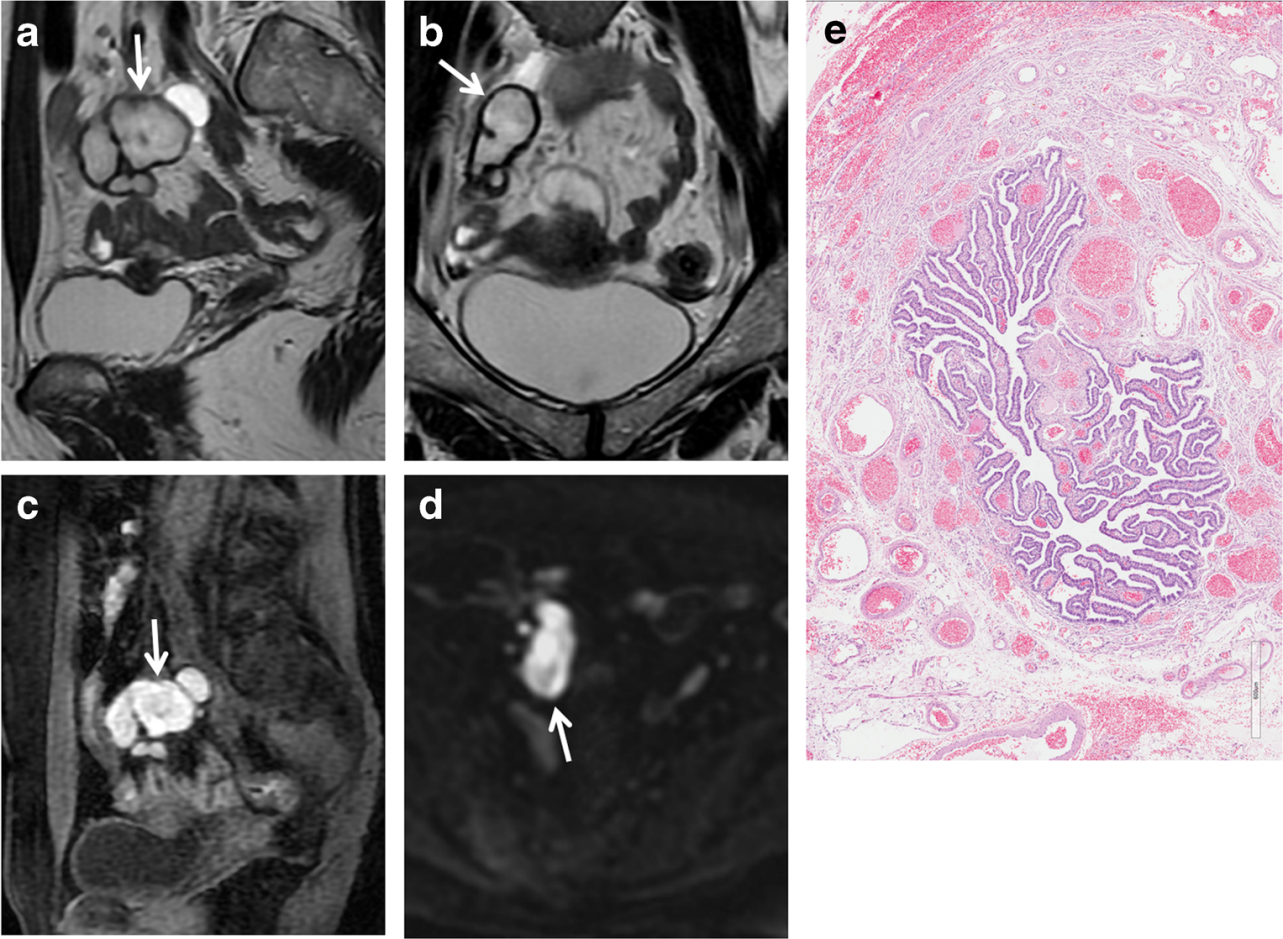
Hematosalpinx. Same patient as in Fig. 5. (**a**) Sagittal and (**b**) coronal T2-weighted MR images show a distended right fallopian tube with dark thick wall and intermediate signal intensity content (*white arrows*). (**c**) Sagittal fat-suppressed T1-weighted image shows a distended fallopian tube with high signal intensity content (*white arrow*), a finding consistent with hematosalpinx (**d**) Axial DW image (b = 800 s/mm^2^) shows that the lesion’s content demonstrates restricted diffusion with high signal intensity (*white arrow*). (**e**) Haematoxylin and eosin stained sample showed massive intramural haemorrhage and vascular ectasia (H&E 150X)

**Fig. 7:**
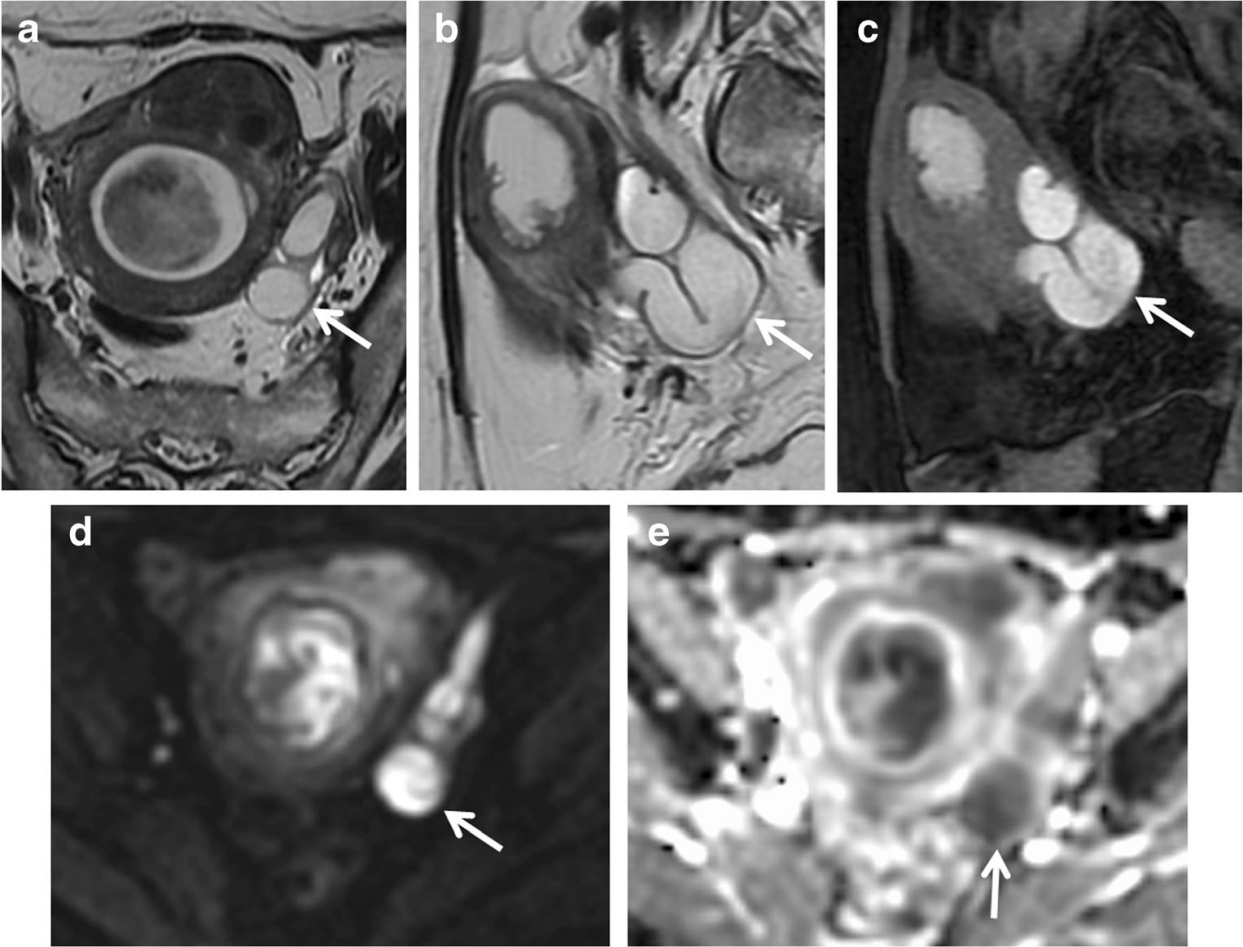
Hematosalpinx in a 62-year-old woman with cervical carcinoma. (**a**) Axial and (**b**) sagittal T2-weighted images show a serpentine fluid-filled left adnexal structure with internal high to intermediate signal intensity (*white arrows*). The endometrial cavity is distended by high to intermediate signal intensity content. (**c**) Sagittal fat-suppressed T1-weighted image show a distended fallopian tube with high signal intensity content (*white arrows*), a finding consistent with hematosalpinx. (**d**) Axial DW image (b = 800 s/mm^2^) and (**e**) corresponding ADC map show that the tubaric content demonstrates restricted diffusion with high signal intensity (*white arrow*) on DWI image and low signal intensity (*white arrow*) on ADC map

**Fig. 8:**
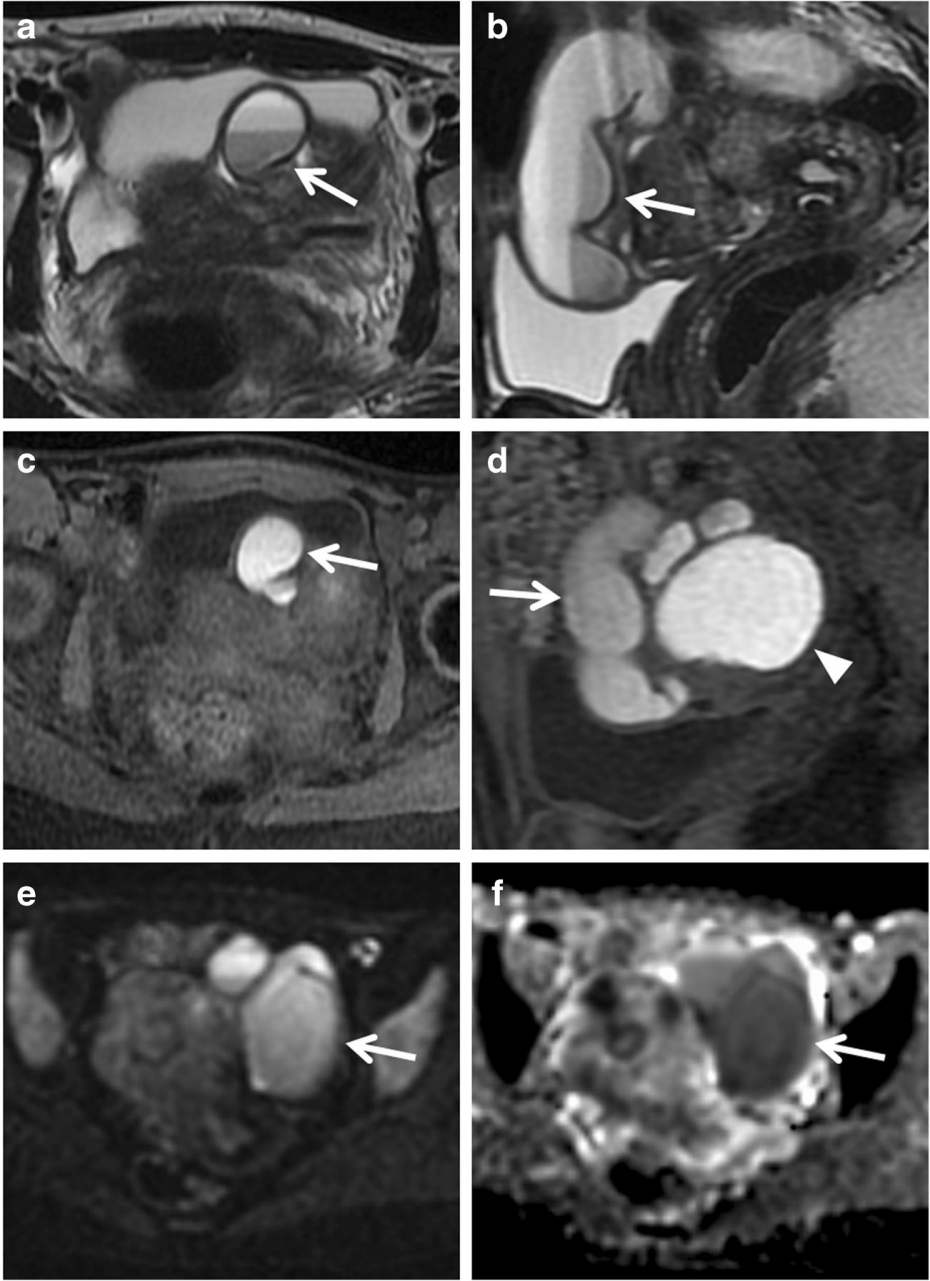
Hematosalpinx in a 35-year-old woman with endometriosis. (**a**) Axial and (**b**) sagittal T2-weighted images show a tortuous, tubular structure with internal fluid-fluid level (white arrows) in the left adnexa. There are incomplete effaced mucosal and submucosal plicae along the tubal wall. (**c**) Axial and (**d**) sagittal fat-suppressed T1-weighted images show a distended fallopian tube with high signal intensity content (*white arrows*), a finding consistent with hematosalpinx. Note an endometriosic cyst in the omolateral ovary (*arrowhead*). (**e**) Axial DW image (b = 800 s/mm^2^) and (**f**) corresponding ADC map show that the lesion’s content demonstrates restricted diffusion with high signal intensity (*white arrow*) on DWI image and low signal intensity (*white arrow*) on ADC map

Hyperintense fluid seen on T1-weighted images is suggestive of *hematosalpinx* (Fig. 6), which is most commonly associated with endometriosis, and more rarely with tubal torsion, tubal ectopic pregnancy or malignancy (Fig. 7). Hematosalpinx appears as a tortuous enlarged tube filled with hemorrhagic fluid [1]. Endometriosis (Fig. 8), characterized by the presence of functional endometrial tissue outside the uterine cavity, is the most common cause of peritubal adhesions in women of reproductive age; in fact, approximately 30 % of women with endometriosis have associated tubal disease that are identified at laparoscopy [53, 54]. Endometrial implants may be serous and subserous, which involves the peritoneal surface of the fallopian tubes, where repeated haemorrhages lead to fibrosis and retraction of the tube with hydrosalpinx. A less common type of tubal endometriosis is intraluminal; in this type, cyclic haemorrhage of the implants can cause hematosalpinx. MR imaging shows hyperintense distention of the fallopian tube on fat-saturated T1- and T2-weighted images. A highly sensitive MR feature of endometiomas is their homogeneous lightbulb-like brightness on T1-weighted fat-suppressed images, which is attributed to the high concentration of paramagnetic haemoglobin in blood breakdown products [55]. Another important feature of the content is “T2 shading”, a feature that ranges from homogeneous signal void through various gradations of decreased signal intensity seen at T2-weighted imaging [56, 57]. It has been reported that endometriosis is a precursor lesion of ovarian malignancies, particularly in endometrioid and clear-cell types. Considering this, caution is needed in precisely confirming the diagnosis of endometrioma, especially in cases with atypical imaging findings. Multilocularity and mural foci or nodules in the hemorrhagic cyst are features associated with malignancy, and contrast material should be administered in these cases. A cystic tumour that is hyperintense on both T1- and T2-weighted images with enhancing mural nodules is often seen in cases of endometriosis complicated by ovarian carcinoma [58–60]. Zhang et al. reported that there isn’t a statistically significant difference in the DWI-MRI signal intensity between endometrioma and malignant tumours [61].

*Fallopian tube torsion* is an exceptionally rare eventuality (1/1.5 million) and usually affects adolescent girls and women of reproductive age [62]. Torsion of the right fallopian tube is much more common, supposedly due to the presence of the sigmoid colon and the mesentery that anchor the tube to the left pelvis. Diagnosis of ovarian torsion continues to be a difficult task, requiring awareness and a high degree of suspicion. The most consistent imaging finding is a unilateral enlarged ovary, without which the diagnosis is unlikely to be ovarian torsion. Comparison with the asymptomatic contralateral side is typically very helpful [63]. MRI is used to differentiate a tubal torsion from ovarian cancer, showing a normal ovary separate from the mass; on T2-weighted images it is seen as a wall thickening of the distended fallopian tube. Sometimes the tube assumes a vortex-like conformation distant from ipsilateral ovary [64]. The dynamic subtraction MR images can also facilitate the demonstration of the absence of blood flow.

The presentation of *acute salpingitis* is the acute phase of the PID in which the fallopian tubes become swollen, edematous, and congested with ascending infection and inflammation. Although not dilated, the swollen fallopian tube may be more conspicuous at imaging due to wall thickening, enhancement, and surrounding inflammation.

**Fig. 9:**
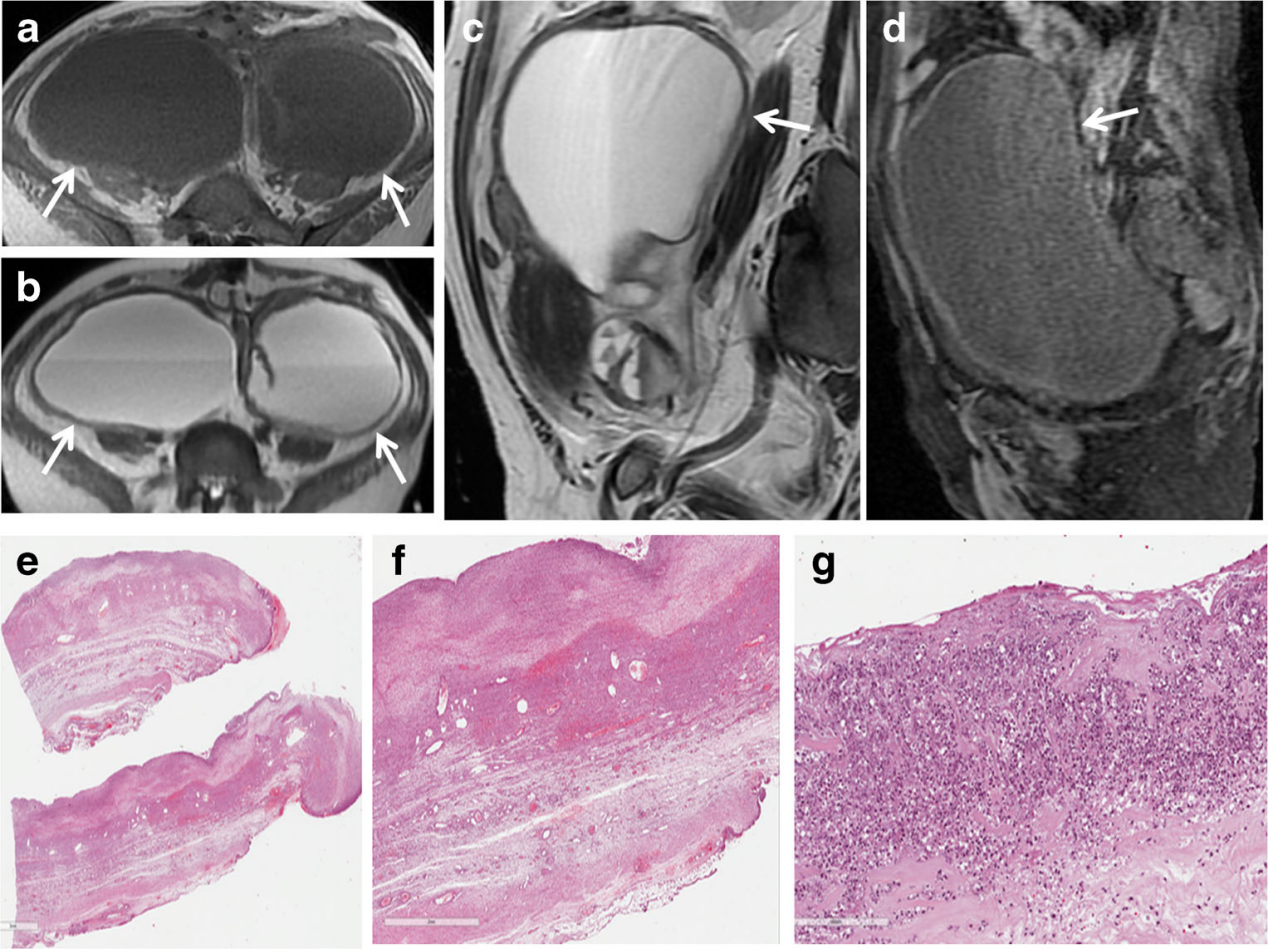
Abscessualized acute salpingitis in a 43-year-old woman who previously underwent hysteroscopy. (**a**) Axial T1-weighted and (**b**) axial T2-weighted images show bilateral sac-like, cystic pelvic masses (*white arrows*) with an internal fluid-debris level. (**c**) Sagittal T2-weighted and (**d**) sagittal fat-suppressed T1-weighted images, show that the pelvis and lower abdominal quadrant are occupied by bulky cystic formations with fluid-corpusculated level content (*white arrows*), better appreciable on T2-weighted MR images. (**e**, **f**, **g**) Histological samples demonstrate the tubal origin of the lesion with broadened and blunted plicae. Numerous leukocytes, lymphocytes, and plasma cells are present in the mucosa (H&E 80X, 150X, 200X)

**Fig. 10:**
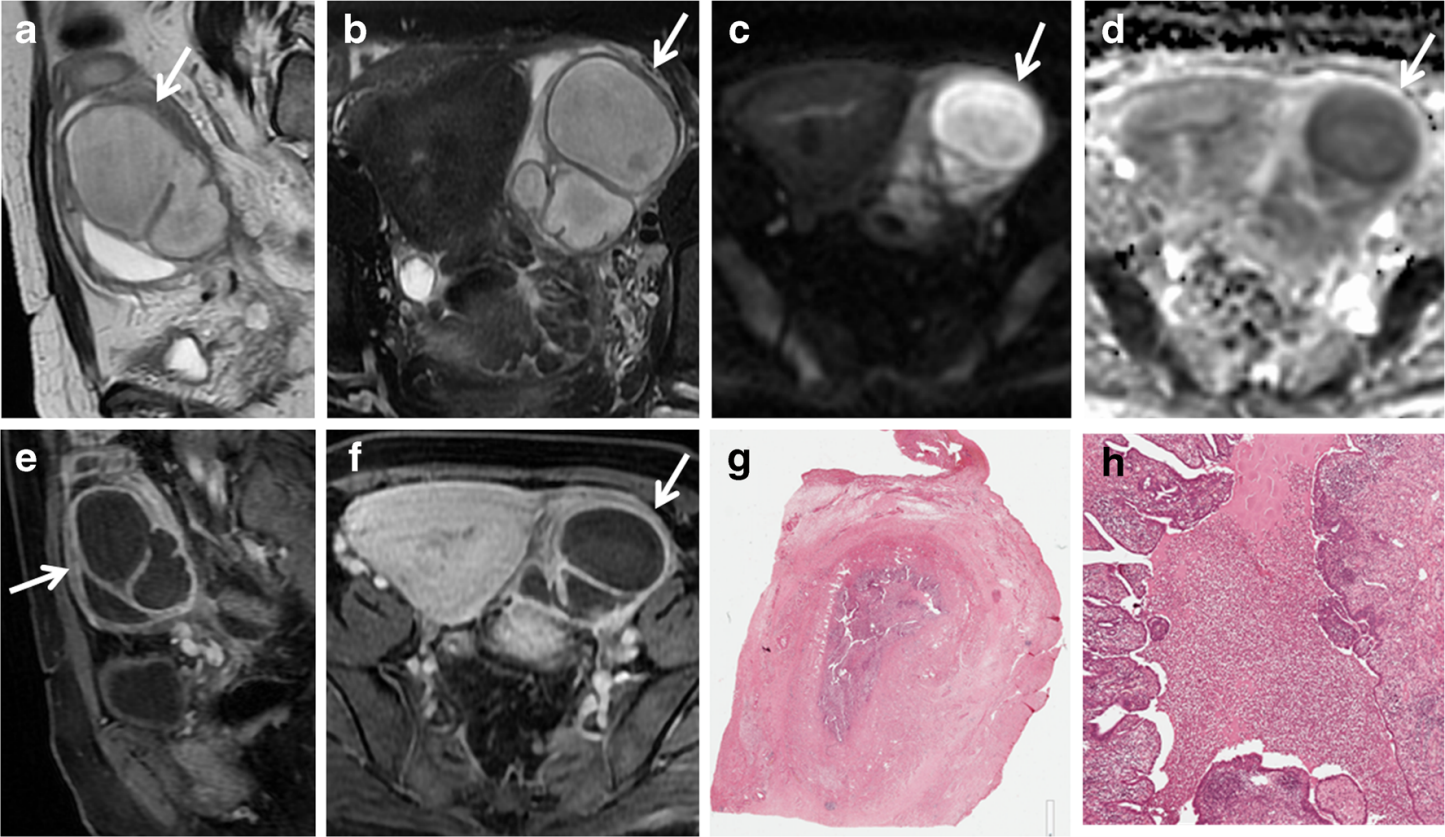
Pyosalpinx in a 47-year-old woman. (**a**) Sagittal T2-weighted and (**b**) coronal fat-suppressed T2-weighted images show distended left fallopian tube with low to intermediate signal intensity content (*white arrows*). (**c**) Axial DW image (b = 800 s/mm^2^) and (**d**) corresponding ADC map show that the lesion’s content demonstrates restricted diffusion with high signal intensity (*white arrow*) on DWI image and low signal intensity (*white arrow*) on ADC map, a finding consistent with purulent content. (**e**) Sagittal and (**f**) coronal contrast-enhanced fat-suppressed T1-weighted images show the thickened, enhancing wall of the dilated tube (*white arrows*). (**g**, **h**) Histologically, leukocytes, histiocytes, few lymphocytes and plasma cells were evident within the wall (H&E 80X). Tubaric lumen showed fibrin strands, leukocytes, and amorphous material (H&E 300X)

**Fig. 11:**
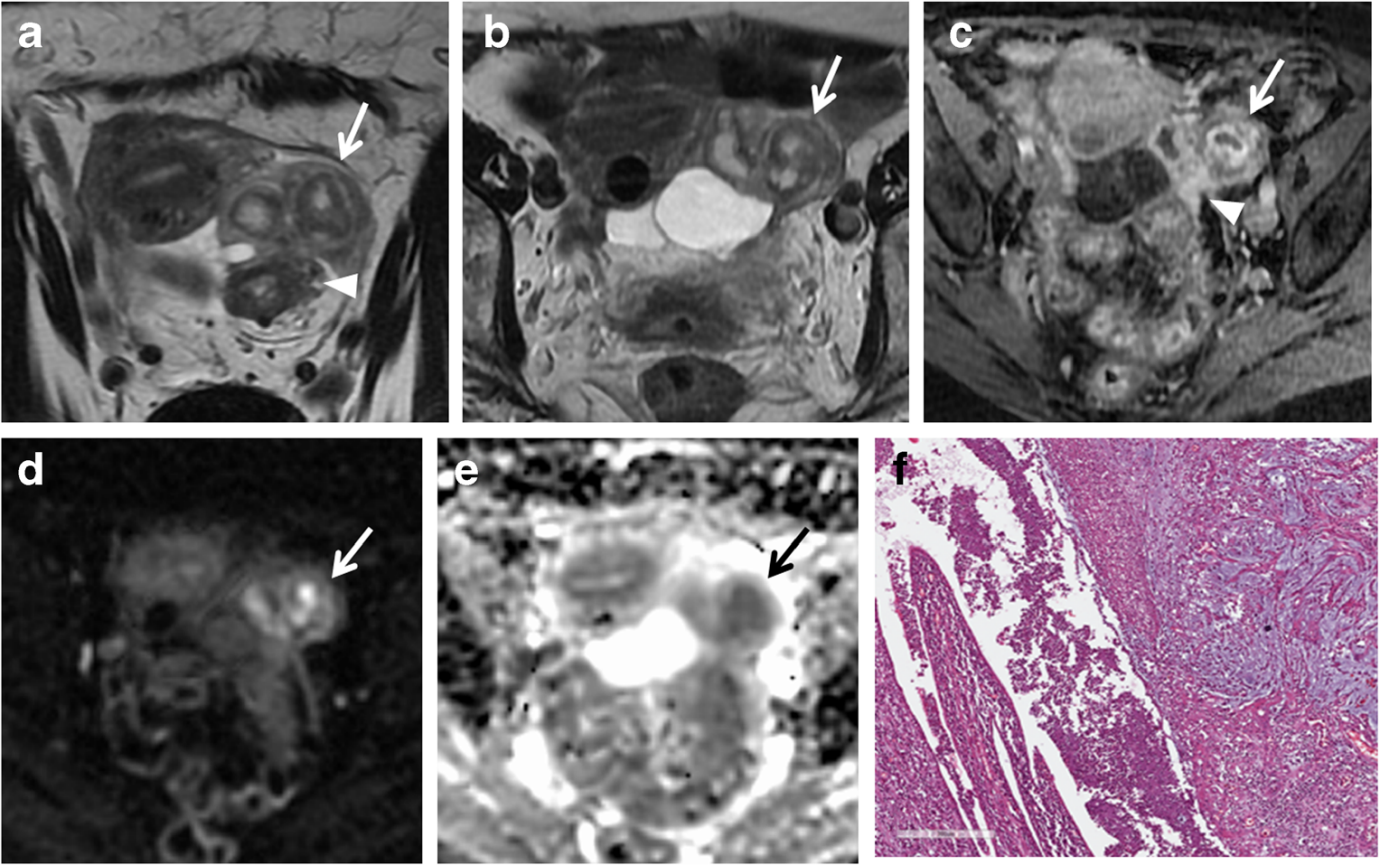
Acute purulent salpingitis in a 62-year-old woman with sigmoid colon diverticulitis. (**a**, **b**) Axial T2-weighted images show a distended left fallopian tube with low to intermediate signal intensity content (*white arrows*); posteriorly the tube is not separable from the sigma, presenting multiple diverticular formations (*white arrowhead* in a). (**c**) Axial contrast fat-suppressed T1-weighted image shows marked enhancement of tubaric wall (*white arrow*) and surrounding fat tissue (*white arrowhead*). (**d**) Axial DW image (b = 800 s/mm^2^) and (**e**) corresponding ADC map show that the lesion’s content demonstrates restricted diffusion with high signal intensity (*white arrow*) on DWI image and low signal intensity (*black arrow*) on ADC map, a finding consistent with a purulent content. (**f**) Histological sample showed purulent material and many leukocytes in the lumen of the tube and confirmed the presence of mucin-containing regular columnar epithelium lining a mucinous cystadenoma (H&E 300X)

A thickened wall of the fallopian tube that shows variable or heterogeneous signal intensity is typical of piosalpinx or tubo-ovarian abscess. *Pyosalpinx* is due to infection with superimposed obstruction of the fallopian tube, is more likely to be bilateral [65], and at MR imaging the tubular structure is readily identified as cystic. The content shows variable signal intensity on T1-weighted images, depending on the protein content of the fluid, but is usually less hyperintense than that observed in hematosalpinx, and is iso-hyperintense on T2-weighted images, with the possible presence of fluid-debris level (Fig. 9). In dubious cases, the enhancement of the thickened fallopian tube walls (Fig. 10), the thickening of the utero-sacral ligament, and oedema of the presacral fat and small-bowel ileum allow a correct diagnosis and differentiation between hydro-and pyosalpinx. In addition, DWI sequences will demonstrate a restriction of diffusion of the walls and purulent content (Fig. 11).

**Fig. 12:**
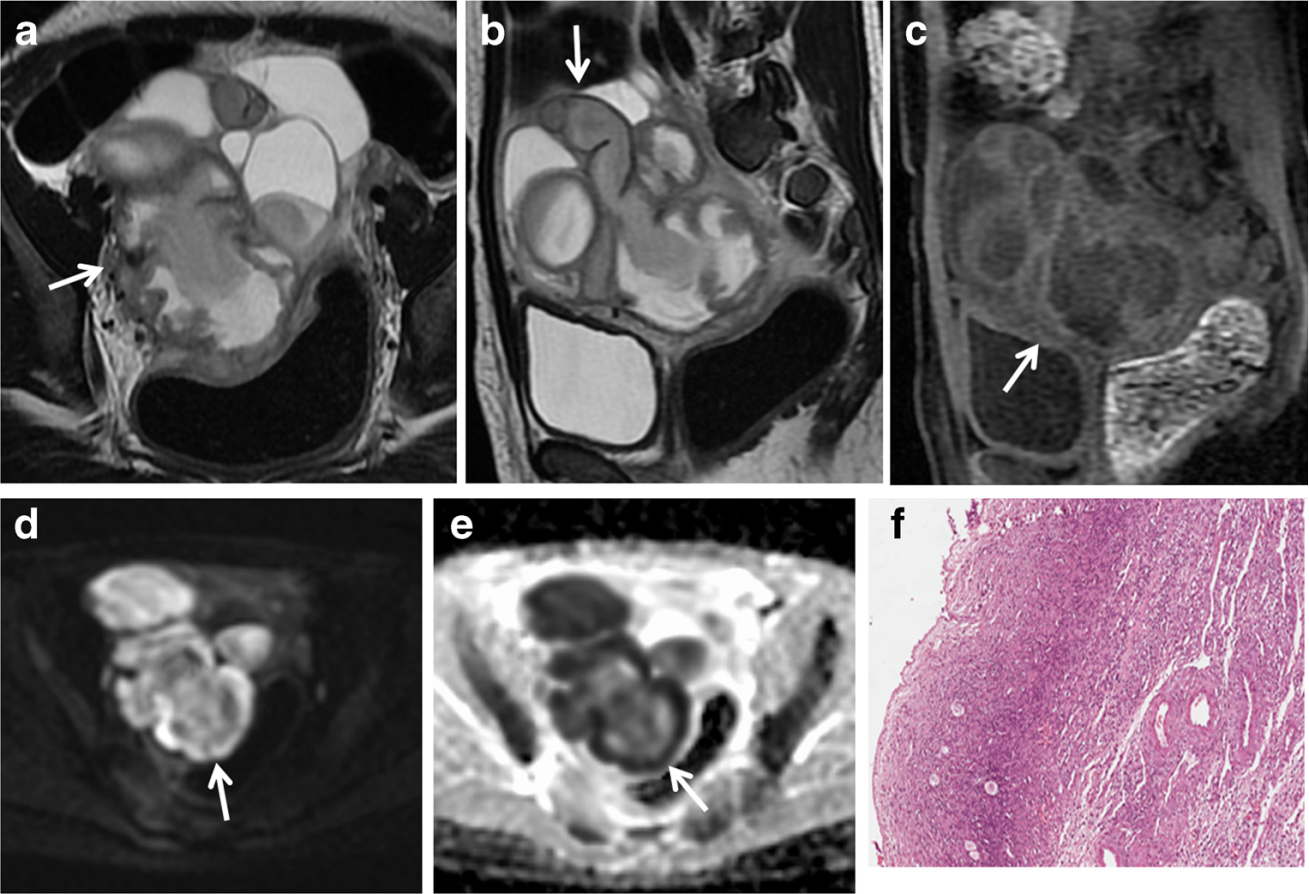
Tubo-ovarian abscess in a 14-year-old woman. (**a**) Axial and (**b**) sagittal T2-weighted images show heterogeneous, multilocular mass, with hypointense internal incomplete plicae and high to intermediate signal intensity fluid content (*white arrows*) in the pelvic cul-de-sac. (**c**) On sagittal fat-suppressed T1-weighted images, the mass’ content shows low signal intensity (*white arrows*). (**d**) Axial DW image (b = 800 s/mm^2^) and (**e**) corresponding ADC map show that the lesion’s content demonstrates restricted diffusion with high signal intensity (*white arrow*) on DWI image and low signal intensity (*white arrow*) on ADC map, a finding consistent with a purulent content. (**f**) The presence of primary ovarian follicles demonstrated the ovarian nature of the lesion (H&E 200X)

**Fig. 13:**
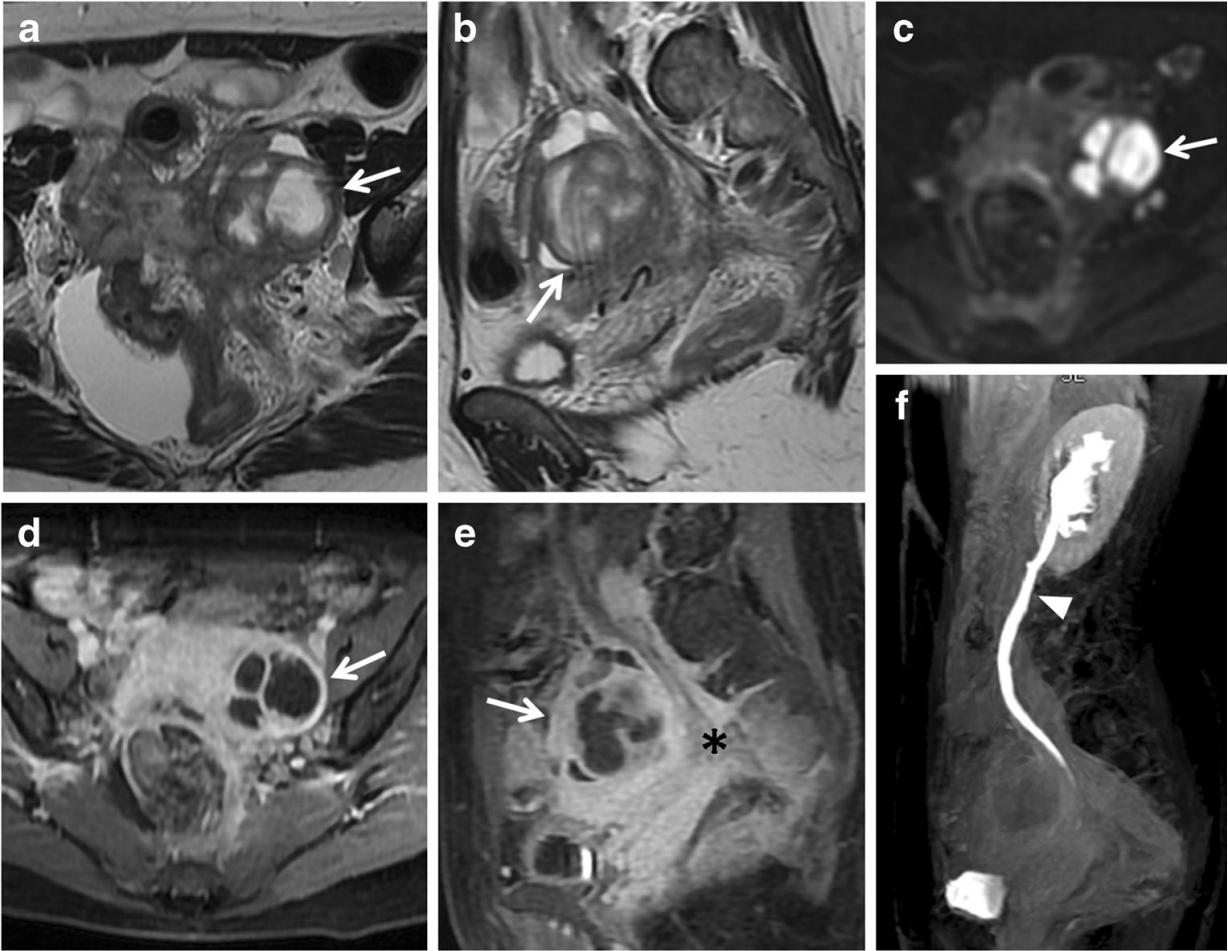
Tubo-ovarian abscess in a 53-year-old woman with acute abdominal pain and elevation of inflammatory markers such as VES and leucocytes. She has used intra-uterine devices (IUDs). (**a**) Axial and (**b**) sagittal T2-weighted images show a multilocular, cystic, pelvic mass with heterogeneous high signal intensity content on T2-weighted images (*white arrows*) in the left side of the pelvis. (**c**) Axial DW image (b = 800 s/mm^2^) demonstrates increased signal of the lesion (*white arrow*), a finding consistent with purulent content. (**d**) Axial and (**e**) sagittal contrast-enhanced fat-suppressed T1-weighted images show septal and thick rim enhancement in the mass (*white arrows*) and in the surrounding inflammatory stranding (*), a finding consistent with tubo-ovarian abscess. (**f**) Oblique sagittal MIP image (acquired about 15 min after contrast administration) shows mild dilatation of the renal pelvis and ureter (*white arrowhead*) due to the compression of tubo-ovarian abscess on the lower third of ureter

Patients with *tubo-ovarian abscess* present with fever and abdominal pain, and the diagnosis is usually made clinically or with transvaginal US. The tubo-ovarian abscess may be due to dissemination of infection by organisms, such as sexually transmitted Chlamydia trachomatis and Neisseria gonorrhoeae, that are causative agents in developing ascending cervicitis with involvement and incorporation of the fallopian tubes and ovaries [41]. Other conditions that may lead to the development of tubo-ovarian abscess are diverticulitis, appendicitis and tuberculosis [41, 66, 67]. It usually appears as a multilocular, cystic pelvic mass with typically low signal intensity on T1-weighted images and heterogeneous high signal intensity on T2-weighted images (Fig. 12); however, hemorrhagic or proteinaceous material can be hyperintense [65, 66, 68]. A thin rim of high signal intensity in the innermost portion of the abscess on T1-weighted images is frequently found and represents a layer of granulation tissue with microscopic haemorrhage [69, 70]. After the intravenous administration of gadolinium-based contrast material, there is septal and thick rim enhancement in the mass and the surrounding inflammatory stranding [69] (Fig. 13). Shading in the peripheral portion of the abscess cavity on T2-weighted images has also been reported as a common finding. Mesh-like strands in the pelvic fat planes due to dense pelvic adhesions or fibrosis are almost always found, and are hypointense on T2-weighted images and well-enhanced [68]. Oedema of the parametrial fat is hyperintense on T2-weighted images [71]. The solid component of the walls and septa of the lesion such as the purulent content exhibit restriction of diffusion on DW images. Tubo-ovarian abscesses frequently cause anterior displacement of the broad ligament, as the mesovarium is positioned more posteriorly, and this can assist in making the diagnosis [67]. The coexistence of tubal dilatation, solid enhancing mass, ascites and enlarged lymph nodes can make it difficult to differentiate the abscess from an ovarian carcinoma [68].

Although radiological features of abscess and ovarian malignancy can be similar, ovarian cancer is not usually associated with tubal dilation [47], and the clinical presentation plays an important role in establishing the diagnosis; follow-up imaging following treatment confirms the decreasing size of adnexal masses in the former case [66, 72].

Imaging findings of primary fallopian tube carcinoma (PFTC) are usually nonspecific, and a tubo-ovarian abscess or ovarian tumour may appear to be the most likely diagnosis given their higher prevalence [47, 73].

Primary fallopian tube carcinoma has a constellation of clinical symptoms and MRI features, which may be diagnostic. Characteristic symptoms of PFTC are: colicky abdominal or pelvic pain, adnexal mass and intermittent, profuse, serosanguineous vaginal discharge, which constitute Laztko’s triad (seen in only 15 % of patients) [47].

Ma et al. identified tubular (sausage) shape, hydrosalpinx, and the presence of intrauterine fluid as the most specific direct and indirect signs of PFTC. The combination of an adnexal mass with at least one of the former features yields a high diagnostic accuracy [47, 74].

A parietal enhancement of a dilated tube surrounded by a cystic mass may eventually indicate a *tubal pregnancy*. Ectopic pregnancy is the main cause of pregnancy-related death during the first trimester in the USA, with an occurrence of 1:150 births [75]. Approximately 95 % of ectopic pregnancies occur in the tubes, with the majority of cases located in the distal parts, particularly the ampulla. When ectopic pregnancy involves the intramural portion of the tube, the highest rate of morbidity and mortality is seen [76]. The other 5 % of cases occur in the ovaries, in a rudimentary horn of the bicornuate uterus, in broad ligaments, in peritoneum, and in the cervix [77]. This event can result from anatomical abnormalities of the tubes, such as constriction and false passage formation (diverticulum), or from tubal dysfunction such as altered contractility or abnormal ciliary activity. Tubal anatomy and function can both be altered by either tubal surgery or prior PID. The surgical procedures that predispose women to ectopic pregnancy include salpingolysis and ovariolysis, fimbrioplasty, neosalpingostomy, and tubal anastomosis.

An ectopic pregnancy should be suspected at any time when a woman of reproductive age with symptoms of acute pelvic pain has positive results of a pregnancy test (or serum β-hCG level above a discriminatory zone of 1,000 to 2,000 mIU/ml) and an intrauterine pregnancy is not definitively seen at imaging [75, 78–82].

**Fig. 14:**
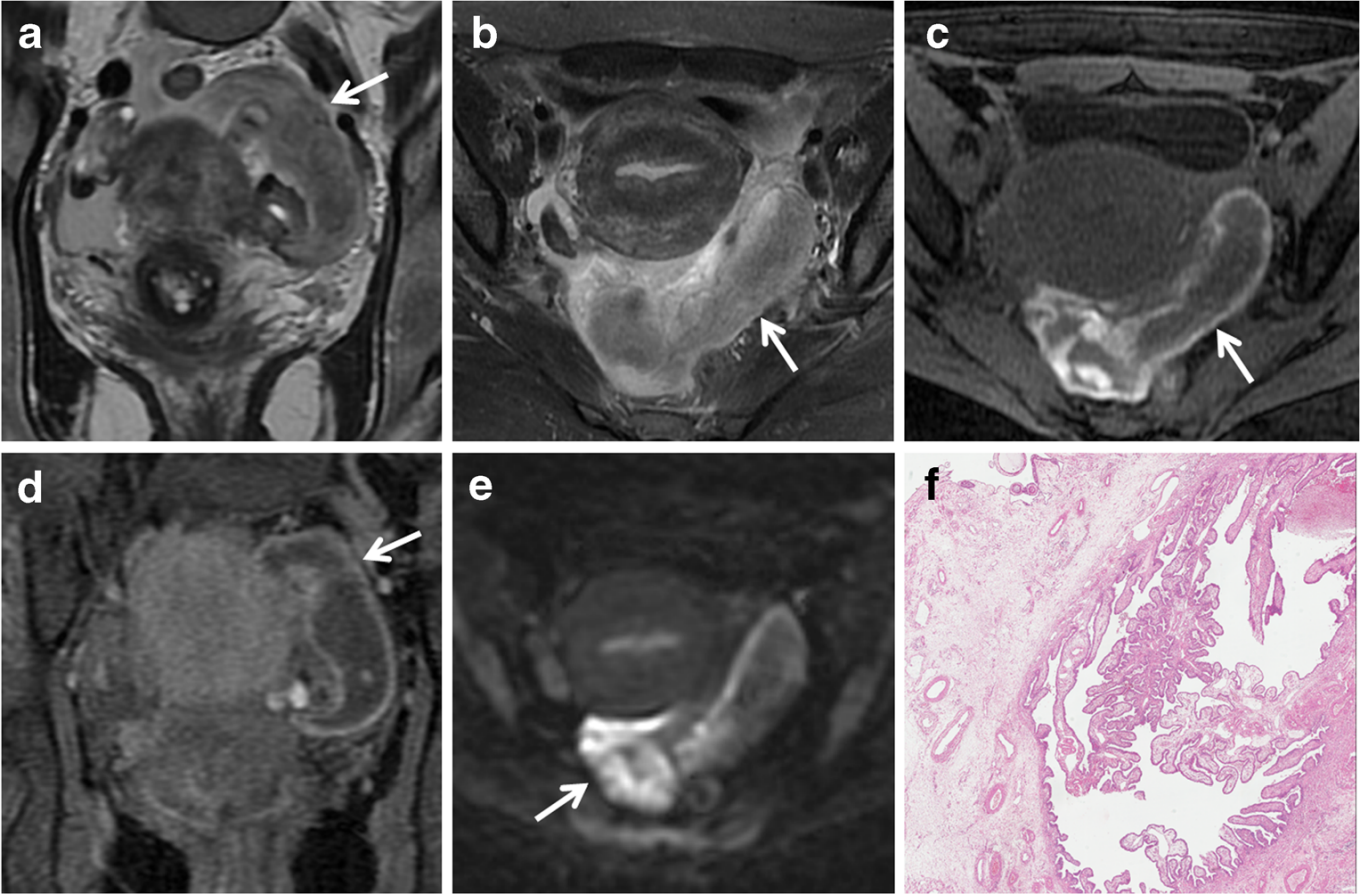
Tubal ectopic pregnancy in a 44-year-old woman with acute abdominal pain and positive results of a pregnancy test (elevated β-hCG level). (**a**) Coronal and (**b**) axial T2-weighted images show a predominantly hypointense saclike cystic adnexal mass (*white arrows*) in the left side of the pelvic cul-de-sac. (**c**) Axial and (**d**) coronal fat-suppressed T1-weighted images show a distended fallopian tube with low to intermediate signal intensity content and high signal intensity thick wall (*white arrows*), a finding consistent with hematosalpinx. (**e**) Axial DW image (b = 800 s/mm^2^) demonstrates increased signal of the lesion (*white arrow*). Note the lack of an intrauterine pregnancy. (**f**) Histologically, villi in the tubal wall confirmed the diagnosis of ectopic tubal pregnancy (H&E 200X)

MR imaging features of an ectopic tubal pregnancy include [7, 38] (Fig. 14):

- hematosalpinx (intermediate signal intensity on T1-weighted images and low signal intensity on T2-weighted images, due to the presence of deoxyhaemoglobin (an acute phase hematoma) [83];
- enhancement of the dilated tube wall (from increased vascularity following implantation) [83];
- presence of a gestational sac (it is seen as a cystic structure surrounded by a thick wall that has intermediate intensity on both T1-weighted with fat suppression and T2-weighted images, and that enhances after administration of contrast material) [2];
- hemorrhagic ascites;
- a heterogeneous adnexal mass;
- lack of an intrauterine pregnancy.

## Conclusions

Developments in MRI techniques have increased the role of MR in diagnosis and characterization of pelvic diseases. Radiologists should be familiar with the imaging features of both normal and pathological appearances of the fallopian tubes in order to allow a correct diagnosis and management of fallopian tube disease. Nowadays, qualitative and quantitative functional imaging with DWI are becoming increasingly important in the evaluation of pelvic disease. DWI is of interest in the detection, characterization and evaluation of responses to treatment of both benign and malignant gynaecologic conditions. To maximize the diagnostic value of DWI, correlation with conventional anatomic MRI sequences is mandatory.

## References

[1] Outwater EK, Siegelman ES, Chiowanich P (1998). Dilated fallopian tubes: MR imaging characteristics. Radiology.

[2] Ascher SM, Edelman RR, Hesselink JR, Zlatkin MB (2006). Benign conditions of the female pelvis. Clinical magnetic resonance imaging.

[3] Thomassin-Naggara I, Daraï E, Cuenod CA, Rouzier R, Callard P, Bazot M (2008). Dynamic contrast-enhanced magnetic resonance imaging: a useful tool for characterizing ovarian epithelial tumors. J Magn Reson Imaging.

[4] Thomassin-Naggara I, Daraï E, Cuenod CA (2009). Contribution of diffusion-weighted MR imaging for predicting benignity of complex adnexal masses. Eur Radiol.

[5] Qayyum A (2009). Diffusion-weighted imaging in th abdomen and pelvis: concepts and applications. Radiographics.

[6] Namimoto T, Awai K, Nakaura T, Yanaga Y, Hirai T, Yamashita Y (2009). Role of diffusion-weighted imaging in the diagnosis of gynecological diseases. Eur Radiol.

[7] Tamai K, Koyama T, Togashi K (2007). MR features of ectopic pregnancy. Eur Radiol.

[8] Tamai K, Koyama T, Saga T (2008). The utility of diffusion-weighted MR imaging for differentiating uterine sarcomas from benign leiomyomas. Eur Radiol.

[9] Shen SH, Chiou YY, Wang JH (2008). Diffusion weighted single-shot echo-planar imaging with parallel technique in assessment of endometrial cancer. AJR Am J Roentgenol.

[10] Tamai K, Koyama T, Saga T (2007). Diffusion-weighted MR imaging of uterine endometrial cancer. J Magn Reson Imaging.

[11] Saremi F, Knoll AN, Bendavid OJ, Schultze-Haakh H, Narula N, Sarlati F (2009). Characterization of genitourinary lesions with diffusion weighted imaging. Radiographics.

[12] http://www.gfmer.ch/International_activities_En/El_Mowafi/Fallopian_tube.htm

[13] Mann G (2012). Imaging of gynecological disorders in infants and children, medical radiology. Diagnostic imaging.

[14] Coleman R, Rao G (2008) Glob. libr. women’s med. doi:10.3843/GLOWM.10262. (ISSN: 1756–2228)

[15] Clemente CD (2007) Anatomy, a regional atlas of the human body. Lippincott Williams & Wilkins. ISBN: 0781751039

[16] Ezzati M, Djahanbakhch O, Arian S, Carr BR (2014). Tubal transport of gametes and embryos: a review of physiology and pathophysiology. J Assist Reprod Genet.

[17] Hill MA (2015) Embryology Uterus Development. Retrieved September 13, 2015, from https://embryology.med.unsw.edu.au/embryology/index.php/Uterus_Development

[18] Sokol E (2011) Glob. libr. women’s med. doi:10.3843/GLOWM.10001. (ISSN: 1756–2228)

[19] http://www.lab.anhb.uwa.edu.au/mb140/corepages/femalerepro/femalerepro.htm

[20] http://teachmeanatomy.info/pelvis/female-reproductive-tract/fallopian-tubes/

[21] Koh DM, Collins DJ (2007). Diffusion-weighted MRI in the body: applications and challenges in oncology. AJR Am J Roentgenol.

[22] Chan JH, Tsui EY, Luk SH (2001). Diffusion-weighted MR imaging of the liver: distinguishing hepatic abscess from cystic or necrotic tumor. Abdom Imaging.

[23] Noguchi K, Watanabe N, Nagayoshi T (1999). Role of diffusion-weighted echoplanar MRI in distinguishing between brain abscess and tumour: a preliminary report. Neuroradiology.

[24] Chavhan GB, Alsabban Z, Babyn PS (2014). Diffusion-weighted imaging in pediatric body MR imaging: principles, technique, and emerging applications. Radiographics.

[25] Neubauer H, Platzer I, Mueller VR (2012). Diffusion-weighted MRI of abscess formations in children and young adults. World J Pediatr.

[26] Stejskal EO, Tanner JE (1965). Spin diffusion measurements: spin-echo in the presence of a time dependent field gradient. J Chem Phys.

[27] Yoshikawa K, Nakata Y, Yamada K, Nakagawa M (2004). Early pathological changes in the parkinsonian brain demonstrated by diffusion tensor MRI. J Neurol Neurosurg Psychiatry.

[28] Eastwood JD, Lev MH, Wintermark M (2003). Correlation of early dynamic CT perfusion imaging with whole-brain MR diffusion and perfusion imaging in acute hemispheric stroke. AJNR Am J Neuroradiol.

[29] Thoeny HC, De Keyzer F (2007). Extracranial applications of diffusion-weighted magnetic resonance imaging. Eur Radiol.

[30] Willinek WA, Schild HH (2008). Clinical advantages of 3.0 T MRI over 1.5 T. Eur J Radiol.

[31] Soher BJ, Dale BM, Merkle EM (2007). A review of MR physics: 3T versus 1.5T. Magn Reson Imaging Clin N Am.

[32] Morakkabati-Spitz N, Gieseke J, Kuhl C (2005). 3.0-T high-field magnetic resonance imaging of the female pelvis: preliminary experiences. Eur Radiol.

[33] Morakkabati-Spitz N, Gieseke J, Kuhl C (2006). MRI of the pelvis at 3 T: very high spatial resolution with sensitivity encoding and flip-angle sweep technique in clinically acceptable scan time. Eur Radiol.

[34] Morakkabati-Spitz N, Schild HH, Kuhl CK (2006). Female pelvis: MR imaging at 3.0 T with sensitivity encoding and flip-angle sweep technique. Radiology.

[35] Zhang H, Zhang GF, He ZY, Li ZY, Zhang GX (2014). Prospective evaluation of 3T MRI findings for primary adnexal lesions and comparison with the final histological diagnosis. Arch Gynecol Obstet.

[36] Saremi F, Jalili M, Sefidbakht S, Channual S, Quane L, Naderi N (2011). Diffusion-weighted imaging of the abdomen at 3 T: image quality comparison with 1.5-T magnet using 3 different imaging sequences. J Comput Assist Tomogr.

[37] Lavdas I, Miquel ME, McRobbie DW, Aboagye EO (2014). Comparison between diffusion-weighted MRI (DW-MRI) at 1.5 and 3 tesla: a phantom study. J Magn Reson Imaging.

[38] Brown MA, Ascher SM, Semelka RC (2006). Adnexa. Abdominal-pelvic MRI.

[39] Rezvani M, Shaaban AM (2011). Fallopian tube disease in the nonpregnant patient. Radiographics.

[40] Yu KK, Hricak H, Higgins CB, Hricak H, Helms CA (1997). The adnexa. Magnetic resonance imaging of the body.

[41] Roche O, Chavan N, Aquilina J, Rockall A (2012). Radiological appearances of gynaecological emergencies. Insights Imaging.

[42] Morse AN, Schroeder CB, Magrina JF, Webb MJ, Wollan PC, Yawn BP (2006). The risk of hydrosalpinx formation and adnexectomy following tubal ligation and subsequent hysterectomy: a historical cohort study. Am J Obstet Gynecol.

[43] Russin LD (1988). Imaging of hydrosalpinx with torsion following tubal sterilization. Semin Ultrasound CT MR.

[44] Abbuhl SB, Muskin EB, Shofer FS (1997). Pelvic inflammatory disease in patients with bilateral tubal ligation. Am J Emerg Med.

[45] Levgur M, Duvivier R (2000). Pelvic inflammatory disease after tubal sterilization: a review. Obstet Gynecol Surv.

[46] Patel MD, Acord DL, Young SW (2006). Likelihood ratio of sonographic findings in discriminating hydrosalpinx from other adnexal masses. AJR Am J Roentgenol.

[47] Veloso Gomes F, Dias JL, Lucas R, Cunha TM (2015). Primary fallopian tube carcinoma: review of MR imaging findings. Insights Imaging.

[48] Ghattamaneni S, Bhuskute NM, Weston MJ, Spencer JA (2009). Discriminative MRI features of fallopian tube masses. Clin Radiol.

[49] Forstner R, Sattlegger P, Heuck A, Reiser M (1998). Abdominal and pelvic MRI.

[50] Whittaker CS, Coady A, Culver L, Rustin G, Padwick M, Padhani AR (2009). Diffusion-weighted MR imaging of female pelvic tumors: a pictorial review. Radiographics.

[51] Punwani S (2011). Diffusion weighted imaging of female pelvic cancers: concepts and clinical applications. Eur J Radiol.

[52] Nougaret S, Tirumani SH, Pandey H, Sala E, Reinhold C (2013). Pearls and pitfalls in MRI of gycologic malignancy with diffusion-weighted technique. AJR Am J Roentgenol.

[53] Ott DJ, Fayez JA, Ott DJ, Fayez JA (1991). Tubal and adnexal abnormalities. Hysterosalpingography: a text and atlas.

[54] Gougoutas CA, Siegelman ES, Hunt J (2000). Pelvic endometriosis: various manifestations and MR imaging findings. AJR Am J Roentgenol.

[55] Lee SI (2006). Radiological reasoning: imaging characterization of bilateral adnexal masses. AJR Am J Roentgenol.

[56] Togashi K, Nishimura K, Kimura I (1991). Endometrial cysts: diagnosis with MR imaging. Radiology.

[57] Glastonbury CM (2002). The shading sign. Radiology.

[58] Tanaka YO, Yoshizako T, Nishida M, Yamaguchi M, Sugimura K, Itai Y (2000). Ovarian carcinoma in patients with endometriosis: MR imaging findings. AJR Am J Roentgenol.

[59] Matsuoka Y, Ohtomo K, Araki T, Kojima K, Yoshikawa W, Fuwa S (2001). MR imaging of clear cell carcinoma of the ovary. Eur Radiol.

[60] Rajkotia K, Veeramani M, Macura KJ (2006). Magnetic resonance imaging of adnexal masses. Top Magn Reson Imaging.

[61] Zhang (2012). Evaluation of primary adnexal masses by 3T MRI: categorization with conventional MR imaging and diffusion-weighted imaging. Journal of Ovarian Research.

[62] Gross M, Blumstein SL, Chow LC (2005). Isolated fallopian tube torsion: a rare twist on a common theme. AJR Am J Roentgenol.

[63] Chang HC (2008). Pearls and pitfalls in diagnosis of ovarian torsion. Radiographics.

[64] Pedrosa I, Zeikus EA, Levine D, Rofsky NM (2007). MR imaging of acute right lower quadrant pain in pregnant and nonpregnant patients. Radiographics.

[65] Horrow MM, Rodgers SK, Naqvi S (2007). Ultrasound of pelvic inflammatory disease. Ultrasound Clin.

[66] Tukeva TA, Aronen HJ, Karjalainen PT, Molander P, Paavonen T, Paavonen J (1999). MR imaging in pelvic inflammatory disease: comparison with laparoscopy and US. Radiology.

[67] Dohke M, Watanabe Y, Okumura A (2000). Comprehensive MR imaging of acute gynecologic diseases. Radiographics.

[68] Ha HK, Lim GY, Cha ES (1995). MR imaging of tubo-ovarian abscess. Acta Radiol.

[69] Kim SH, Kim SH, Yang DM, Kim KA (2004). Unusual causes of tubo-ovarian abscess: CT and MR imaging findings. Radiographics.

[70] Imaoka I, Wada A, Kaji Y, Hayashi T, Hayashi M, Matsuo M (2006). Developing an MR imaging strategy for diagnosis of ovarian masses. Radiographics.

[71] Singh A, Danrad R, Hahn PF, Blake MA, Mueller PR, Novelline RA (2007). MR imaging of the acute abdomen and pelvis: acute appendicitis and beyond. Radiographics.

[72] Sam JW, Jacobs JE, Birnbaum BA (2002). Spectrum of CT findings in acute pyogenic pelvic inflammatory disease. Radiographics.

[73] Kim MY, Rha SE, Oh SN (2009). MR imaging findings of hydrosalpinx: a comprehensive review. Radiographics.

[74] Ma FH, Cai SQ, Qiang JW (2015). MRI for differentiating primary fallopian tube carcinoma from epithelial ovarian cancer. J Magn Reson Imaging.

[75] Masselli G, Brunelli R, Monti R, Guida M, Laghi F, Casciani E (2014). Imaging for acute pelvic pain in pregnancy. Insights Imaging.

[76] Malinowski A, Bates SK (2006). Semantics and pitfalls in the diagnosis of cornual/interstitial pregnancy. Fertil Steril.

[77] http://www.gfmer.ch/Obstetrics_simplified/Ectopic_pregnancy.htm.

[78] Parker RA, Yano M, Tai AW, Friedman M, Narra VR, Menias CO (2012). MR imaging findings of ectopic pregnancy: a pictorial review. Radiographics.

[79] Cacciatore B (1990). Can the status of tubal pregnancy be predicted with transvaginal sonography? a prospective comparison of sonographic, surgical, and serum hCG findings. Radiology.

[80] Goldstein SR, Snyder JR, Watson C, Danon M (1988). Very early pregnancy detection with endovaginal ultrasound. Obstet Gynecol.

[81] Nyberg DA, Mack LA, Laing FC, Jeffrey RB (1988). Early pregnancy complications: endovaginal sonographic findings correlated with human chorionic gonadotropin levels. Radiology.

[82] Doubilet PM, Benson CB (2011). Further evidence against the reliability of the human chorionic gonadotropin discriminatory level. J Ultrasound Med.

[83] Kataoka ML, Togashi K, Kobayashi H (1999). Evaluation of ectopic pregnancy by magnetic resonance imaging. Hum Reprod.

